# In Vivo Molecular Dissection of the Effects of HIV-1 in Active Tuberculosis

**DOI:** 10.1371/journal.ppat.1005469

**Published:** 2016-03-17

**Authors:** Lucy C. K. Bell, Gabriele Pollara, Mellissa Pascoe, Gillian S. Tomlinson, Rannakoe J. Lehloenya, Jennifer Roe, Richard Meldau, Robert F. Miller, Alan Ramsay, Benjamin M. Chain, Keertan Dheda, Mahdad Noursadeghi

**Affiliations:** 1 Division of Infection and Immunity, University College London, London, United Kingdom; 2 Lung Infection and Immunity Unit, Division of Pulmonology and UCT Lung Institute, Department of Medicine, University of Cape Town, Cape Town, South Africa; 3 Institute of Epidemiology and Healthcare, University College London, London, United Kingdom; 4 Cellular Pathology, University College Hospitals London, London, United Kingdom; Harvard School of Public Health, UNITED STATES

## Abstract

Increased risk of tuberculosis (TB) associated with HIV-1 infection is primarily attributed to deficient T helper (Th)1 immune responses, but most people with active TB have robust Th1 responses, indicating that these are not sufficient to protect against disease. Recent findings suggest that favourable outcomes following *Mycobacterium tuberculosis* infection arise from finely balanced inflammatory and regulatory pathways, achieving pathogen control without immunopathology. We hypothesised that HIV-1 and antiretroviral therapy (ART) exert widespread changes to cell mediated immunity, which may compromise the optimal host protective response to TB and provide novel insights into the correlates of immune protection and pathogenesis. We sought to define these effects in patients with active TB by transcriptional profiling of tuberculin skin tests (TST) to make comprehensive molecular level assessments of in vivo human immune responses at the site of a standardised mycobacterial challenge. We showed that the TST transcriptome accurately reflects the molecular pathology at the site of human pulmonary TB, and used this approach to investigate immune dysregulation in HIV-1/TB co-infected patients with distinct clinical phenotypes associated with TST reactivity or anergy and unmasking TB immune reconstitution inflammatory syndrome (IRIS) after initiation of ART. HIV-1 infected patients with positive TSTs exhibited preserved Th1 responses but deficient immunoregulatory IL10-inducible responses. Those with clinically negative TSTs revealed profound anergy of innate as well as adaptive immune responses, except for preservation of type 1 interferon activity, implicated in impaired anti-mycobacterial immunity. Patients with unmasking TB IRIS showed recovery of Th1 immunity to normal levels, but exaggerated Th2-associated responses specifically. These mechanisms of immune dysregulation were localised to the tissue microenvironment and not evident in peripheral blood. TST molecular profiling categorised different mechanisms of immunological dysfunction in HIV-1 infection beyond the effects on CD4 T cells, each associated with increased risk of TB disease and amenable to host-directed therapies.

## Author Summary

HIV-1 infected people have substantially increased risk of tuberculosis (TB) leading to a large burden of disease worldwide. We aimed to investigate how HIV-1 causes this effect by altering human immune responses. We measured the products of all immune genes at injection sites of sterilized TB under the skin, in order to look for differences between TB patients with and without HIV-1. We found that the predominant effect of early HIV-1 infection was to diminish a component of immune responses that contributes to prevention of harmful inflammation. In more advanced HIV-1, we found almost complete absence of any immune response to TB except for immune activity which is normally part of our defence against viruses, but may also weaken immune protection against TB. In some patients, TB becomes apparent after starting treatment for HIV-1. In these patients we found that most immune responses had recovered to normal levels, but that one type of response sometimes associated with asthma and allergies was exaggerated. Our findings provide new insights into how HIV-1 can affect immune responses and changes to the immune system that are associated with risk of TB, which will inform the development of new strategies to improve protective immunity.

## Introduction

One and a half million deaths are attributed to nine million new cases of active tuberculosis (TB) per annum [1]. Most individuals infected with *Mycobacterium tuberculosis* (Mtb) do not develop disease, but co-infection with Human immunodeficiency virus (HIV)-1 substantially increases this risk, even before progression to advanced acquired immunodeficiency syndrome (AIDS) [2,3]. HIV-1 associated TB presents more frequently as primary infection and extrapulmonary or disseminated disease [4], suggesting inadequate immunological control of Mtb. In addition, rare genetic immunodeficiencies show unequivocally that interferon (IFN)γ responses and signalling pathways associated with CD4 T helper (Th)1 immunity are necessary for protection against mycobacterial infection generally [5]. However, most people with active TB typically exhibit robust Th1/IFNγ responses [6,7] that may even contribute to immunopathology [8,9]. Therefore, factors other than Th1 immunity must contribute to protection. Recent findings suggest that favourable outcomes following Mtb infection arise from finely balanced inflammatory and regulatory pathways, and point to a putative detrimental role for type 1 IFNs [10–13]. Investigation of HIV-1 associated TB has focussed on deficient CD4 T cell responses which are evident before severe depletion of circulating CD4 T cells in AIDS [14]. However, HIV-1 infection also causes persistent type 1 IFN responses and chronic immune activation by diverse mechanisms [15–17]. Therefore, increased TB disease in HIV-1 infected patients may arise as a result of inadequate inflammatory responses that are unable to control bacillary growth, or exaggerated inflammatory responses that lead to increased immunopathogenesis. The latter are widely implicated in the mechanism underlying TB immune reconstitution inflammatory syndrome (IRIS) which can occur after initiating treatment for HIV-1 with antiretroviral therapy (ART) [18,19]. We hypothesise that unbiased genome-wide assessments of anti-mycobacterial immune responses in HIV-1 patients with and without IRIS may identify deficient responses that contribute to host protection against TB, or exaggerated responses that drive its pathogenesis. These may also extend our general understanding of immunological correlates of protection and pathogenesis in TB, and thereby allow better stratification of the risk of disease after Mtb infection, rational design of novel vaccines and development of host-directed therapies to radically shorten duration of treatment or mitigate against increasing TB drug resistance [20,21].

Systems level assessments of human immunobiology in active TB have principally described changes in peripheral blood transcriptional profiles. Quantitatively these changes correlate with disease severity and resolve with treatment [13,22,23]. Therefore, differences between patient populations in cross-sectional studies are likely to be confounded by differences in the bacillary burden and duration of infection, neither of which can be measured accurately. Moreover, recent longitudinal functional imaging studies in experimental non-human primate models and in patients with active TB revealed that different foci of disease within the same host show autonomous and discordant activity [24–26], indicating localised immunopathology within the tissue microenvironment which may not be represented faithfully by measurements in the systemic circulation. In order to address these limitations, we undertook whole genome transcriptional profiling of biopsies from the tuberculin skin test (TST) in order to make comprehensive molecular level assessments of human immune responses to TB at the site of a standardised experimental challenge. Clinical inflammation in response to the TST has been widely used as a measure of anti-mycobacterial T cell memory [27,28], but we have previously shown that transcriptional profiling of the TST in healthy people provides quantitative and qualitative measurements of multivariate molecular components of innate and adaptive cell mediated immunity [29]. Importantly, we were also able to detect changes in transcript abundance in the absence of clinical inflammation, and thereby identify immune responses in TSTs that would otherwise be described as anergic by conventional assessments. This offers unprecedented opportunities to assess immune responses to TB in patients with advanced HIV-1 infection who frequently exhibit clinically negative TST responses [30].

In this study we demonstrate that transcriptional profiling of the TST in patients with active TB accurately models molecular pathology within foci of pulmonary TB. We use this approach to identify changes to in vivo human immune responses to TB at different stages of HIV-1 infection in patients with clinically positive or negative TSTs and in patients with unmasking TB IRIS, each group representing different immunological states associated with increased risk of active TB or increased immunopathology. By taking a genome-wide approach, we test the hypothesis that the dysregulation of anti-mycobacterial responses in each of these states extends beyond selective deficiency or exaggeration of Th1 responses to other immunological components that may also contribute to increased risk of TB.

## Results

### Molecular profiling of the TST in HIV-1 negative patients with active TB

**Fig 1:**
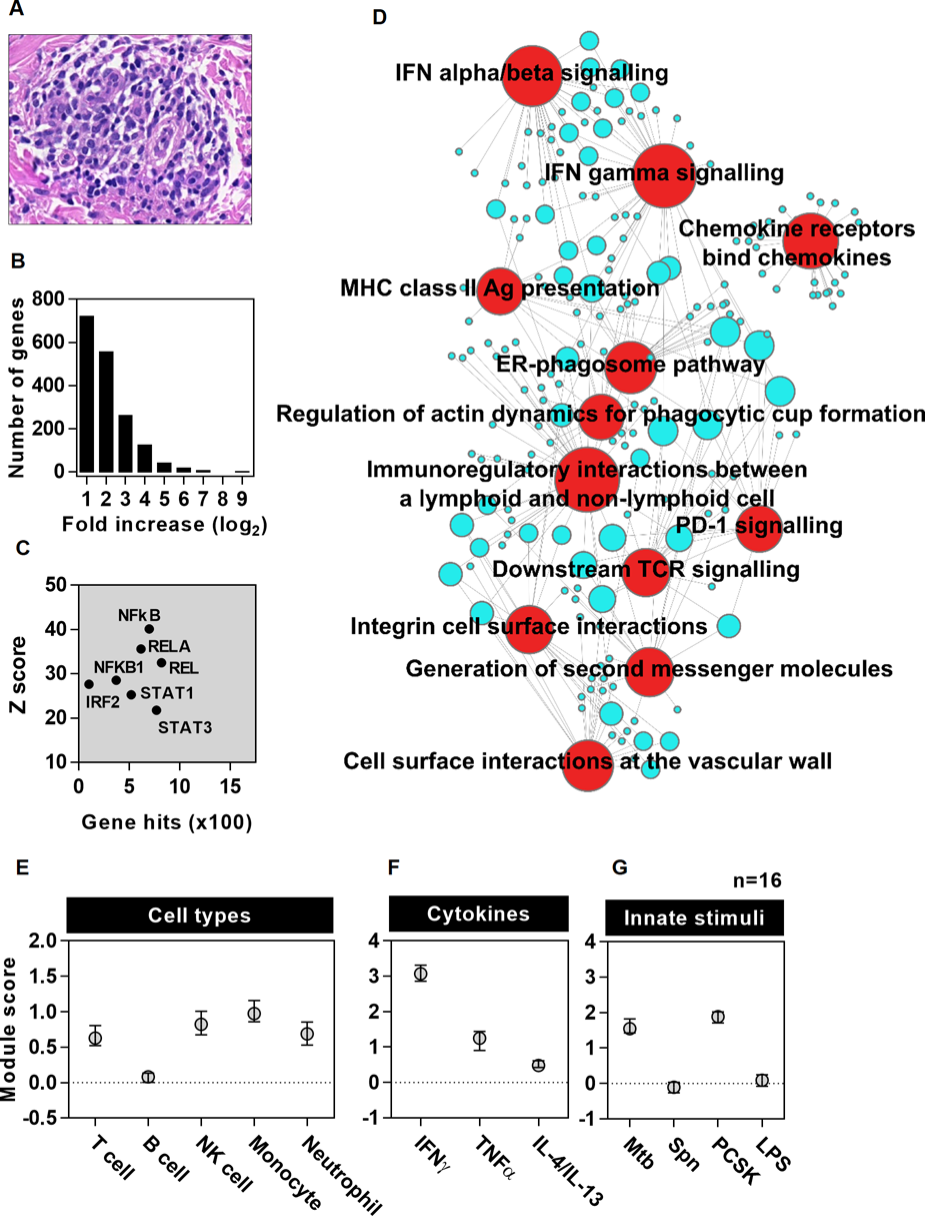
Bioinformatic and modular analysis of the TST signature in HIV negative patients with active TB. (a) Representative example of histological leukocytic infiltrate associated with a clinically positive TST. (b) Frequency distribution of median fold increases in TST transcript abundance in HIV-1 seronegative patients with active TB (n = 16) compared to site of saline injections (n = 8). TFBS enrichment analysis for this gene list is shown indicating the number of genes and statistical enrichment (Z score) associated with selected transcription factors (c). Associations with top twelve most statistically enriched REACTOME functional pathways are represented in a network plot (d), in which the edges indicate associations between genes (blue nodes) and named pathways (red nodes), and the node size is proportional to the number of associations. (e-g) Module scores represent log ratio of geometric mean of gene expression for each module within TST compared to saline biopsies (data points represent median ±IQR).

We first sought to describe the response to 48 hour TSTs in HIV seronegative patients with active TB as early as possible after diagnosis and within one month of starting anti-mycobacterial treatment. The TST caused clinically evident inflammation and histology revealed leukocytic infiltrates consistent with a cell mediated immune response (Fig 1A and S1 Fig). A TST transcriptional signature was identified, comprising 1725 genes with significantly increased transcript abundance compared to biopsies of control saline injections (Fig 1B). Transcription factor binding site (TFBS) enrichment analysis was used to infer upstream regulation of the TST gene signature. Binding sites for NFκB family members showed most statistically significant enrichment, but enrichment of IRF2, STAT1 and STAT3 binding sites were also evident, consistent with innate immune, IFN and other cytokine stimulated signalling associated with each of these transcription factors (Fig 1C and S1 File). Pathway analysis of this gene set confirmed well established components of cell mediated immunity including enrichment of chemokine activity, antigen presentation, T cell activation and IFN signalling (Fig 1D, S2 File). Although peripheral blood transcriptional changes in active TB have been reported within the first week of treatment [23], differences in the duration of treatment of individual patients in the present study were not associated with segregated clustering of TST transcriptional signatures in principal component analysis (PCA) (S3A Fig). Likewise, the TST transcriptional profile of patients with pulmonary and extrapulmonary TB clustered together in the PCA (S3B Fig), indicating that neither time on treatment within one month, nor site of active TB disease, caused substantial confounding of the transcriptional response to tuberculin challenge.

In order to validate and extend these analyses, we derived predefined cell-type and stimulus-specific gene modules (S3 and S4 Files) from independent experimental data to investigate the relative enrichment of each module within the genome-wide transcriptome of TSTs compared to control saline injections. Cell type specific modules were derived by comparison of transcriptomes from selected purified immune cells (S4 and S5A Figs), and independently validated in data sets from purified cell types and diverse tissue specimens in which module enrichment was consistent with histological assessments of cellular composition (S5B–S5G Fig). Cytokine or innate immune stimulus-specific modules were derived from differential transcriptional responses of monocyte derived macrophages (MDM) to selected cytokines associated with alternatively polarised T cell function and to selected innate immune stimuli associated with Mtb or alternative pathogens (S6 Fig). Analysis of cellular modules showed selective accumulation of circulating leukocytes within the TST. T and NK cell, monocyte and neutrophil derived modules were most enriched, but there was significantly less enrichment of the B cell module (Fig 1E). Cytokine specific gene modules showed greatest enrichment of IFNγ inducible transcripts, which we infer to predominantly reflect Th1 activity. Some enrichment of TNFα activity, which is also thought to contribute to protection to immune protection in TB [31], and IL4/IL13 activity associated with Th2 polarised responses [32] were also evident to a lesser degree (Fig 1F). Innate immune specific modules showed enrichment of genes exclusively upregulated by Mtb and TLR2 stimulation, but not genes exclusively upregulated by TLR4 or *Streptococcus pneumoniae* (Spn) (Fig 1G). Although TLR2 is a well-established innate immune receptor for both Mtb and Spn, TLR2 independent responses to Spn are also recognised [33,34]. Therefore, we interpret the absence of any modular enrichment for TLR4 and Spn exclusive responses as evidence for specific anti-mycobacterial innate immune responses within the TST rather than non-specific inflammation. In order to confirm that differences in duration of treatment or site of disease did not confound the TST transcriptome at systems level, we also showed that neither of these variables affected the expression of cell type or stimulus specific modules (S7 Fig).

**Fig 2:**
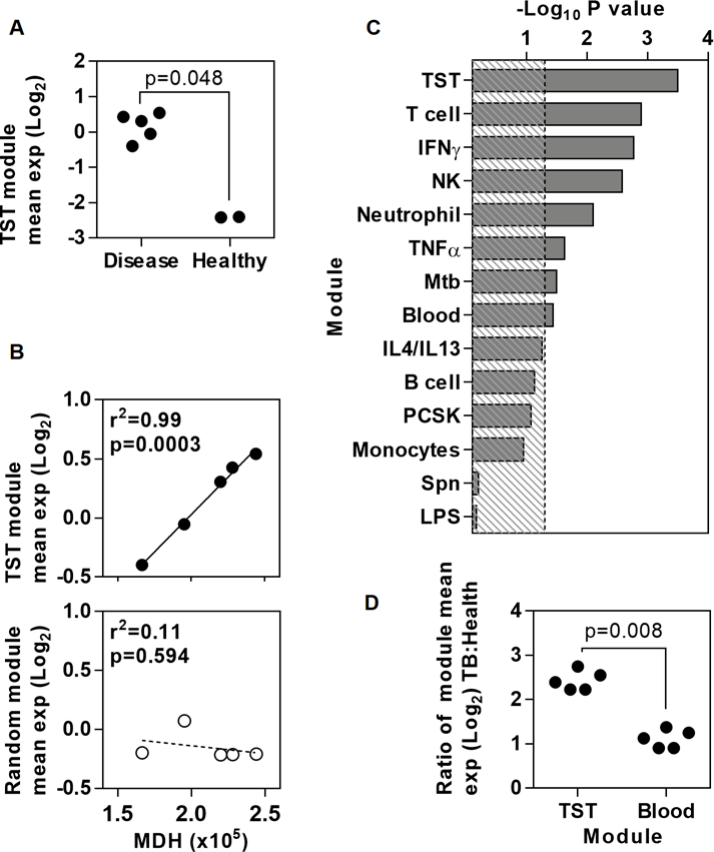
Expression of the TST signature genes in granuloma at the site of disease in pulmonary TB. Relative mean expression of the TST gene signature from HIV-1 negative patients with active TB, or equivalent number of random genes, in transcriptional data from human lung TB granuloma (n = 5) is compared to (a) data from healthy lung (n = 2), and to (b) genome-wide MDH in TB granulomas. (c) The statistical strength of correlation between mean expression of specific gene modules and genome-wide MDH in TB granulomas. Dotted line indicates–log_10_ p value of 1.3 equivalent to p<0.05. (d) Relative enrichment of mean expression of the TST and peripheral blood gene signatures from HIV-1 negative patients with active TB in TB granulomas compared to healthy lung. Individual data points are shown in (a), (b) and (d). P values in (a) and (d) are derived from Mann-Whitney tests, and in (b-c) are derived from Pearson’s correlations.

In order to test the validity of the TST as a model for TB immunopathology, we next assessed the expression of the TST signature within published genome-wide transcriptomes of human lung TB granulomas and healthy human lung [35]. The geometric mean expression for the TST gene signature was greater within TB granulomas (Fig 2A) and correlated precisely with the magnitude of genome-wide transcriptomic differences between granulomatous TB and healthy lung tissue, which was represented by molecular distance to health (MDH) for each of the granulomatous lesions. In contrast, a signature of the same number of randomly selected genes showed no correlation (Fig 2B). Moreover, the correlation between MDH and the geometric mean of the TST signature was statistically stronger than that of any other cell or stimulus specific gene expression module (Fig 2C), suggesting that the TST signature better reflects variability at the site of disease than any of its component parts. Changes in peripheral blood transcriptomes of patients with active TB have also been associated with severity of clinical disease [13]. For comparison with the TST signature, we derived a peripheral blood transcriptional signature of genes with significantly increased expression levels in our patients with active TB compared to healthy volunteers, which also showed statistically significant correlation with MDH in pulmonary TB granulomas (S8 Fig, Fig 2C). The blood derived signature showed some overlap with the TST signature at molecular and systems levels (S8 Fig). Importantly, however, the TST transcriptional signature included immune response genes that were not increased in the blood of patients with active TB. Notably, these included components of chemokine networks that can be expressed by endothelial cells [36,37] within tissue biopsy specimens and may contribute to immune cell recruitment (S8 Fig). Moreover, granulomatous inflammation in response to TB, which is dependent on immune cell recruitment, occurs exclusively in tissues rather than blood. Accordingly, within pulmonary TB granulomas, the TST signature was significantly more enriched and showed a statistically stronger correlation with MDH than the blood derived signature (Fig 2C and 2D). Taken together, these analyses show that the transcriptional changes within the TST reflect molecular changes at the site of active TB with much higher fidelity than changes in peripheral blood, and can be used to quantify cellular recruitment and specific innate and adaptive immune responses.

### HIV-1 attenuation of IL10 responses to TST in patients with positive TST

We hypothesised that HIV-1 infection may have distinct effects on the immune response to TB at different stages of HIV-1 disease. Therefore, we evaluated TST responses in three separate clinical phenotypes comprising patients with active TB and HIV-1 co-infection who either exhibited a clinically positive TST response, or those who exhibited a clinically negative TST response, or patients with possible unmasking TB-IRIS (Table 1 and S1 Table). The TST transcriptome of each group was compared to that of HIV seronegative patients with active TB, as a standard reference. We focussed on patients with active TB to overcome the difficulty in controlling for TB exposure in asymptomatic HIV-1 positive individuals for whom HIV-1 infection confounds peripheral blood interferon gamma release assays normally used to identify prior exposure [28,38,39]. In addition, this approach allows assessment of immune dysfunction associated with IRIS. In view of the greatly increased risk of active TB in HIV-1 infection, and the increase in immunopathogenesis in IRIS, we hypothesised that features in the TST transcriptomes that were decreased in each of the groups of HIV-1 infected patients may represent immune correlates of protection, and that features that were increased may represent immune correlates of pathogenesis.

**Table 1:** Summary of demographic, clinical and laboratory data of study groups.

Participant characteristics		Patients with active TB						HIV -ve healthy volunteers
		TST				Saline controls		
		HIV -ve	HIV +ve			HIV -ve	HIV +ve	TST -ve
			TST -ve	TST +ve	TB-IRIS			
Number		16	14	9	3	3	5	5
Age (median & range)		38 (25–71)	36.5 (26–58)	40 (23–64)	37 (19–50)	45 (29–54)	39 (30–53)	36 (22–44)
Gender	Male (%)	62.50	50	33.33	0	66.67	60	40
	Female (%)	37.50	50	66.67	100	33.33	40	60
Ethnicity	White (%)	12.50	0	0	0	0	0	20
	Black (%)	62.50	92.86	100	100	66.67	80	0
	Mixed (%)	0	7.14	0	0	33.33	20	80
	Asian (%)	25	0	0	0	0	0	0
Site of disease	Pulmonary (%)	56.25	71.43	100	100	100	80	N/A
	Extra-pulmonary (%)	43.75	28.57	0	0	0	20	
MDR TB (%)		12.50	0	0	0	0	0	N/A
TB treatment days at TST (median & range)		11.5 (1–32)	9 (1–28)	15 (2–28)	10 (3–13)	4 (3–4)	2 (1–13)	N/A
CD4 count (cells/μL, median & range)		N/A	28 (2–207)	214 (34–511)	279 (128–507)	N/A	105 (46–412)	N/A
Log_10_ HIV-1 copies/mL (median & range)		N/A	5.3 (2.1–6.3)	5.2 (1.7–6.0)	1.8 (1.7–5.3)	N/A	4.8 (1.7–5.9)	N/A
ARV use (%)		N/A	35.7	55.56	100	N/A	40.00	N/A
Weeks ART before TB (median & range)		N/A	0.7 (0–24)	123 (0–311)	3.7 (2–6.6)	N/A	2–6.6	N/A
mm induration (median & range)		21 (12–28)	0	16 (10–24)	24 (21–26)	N/A	N/A	0

**Fig 3:**
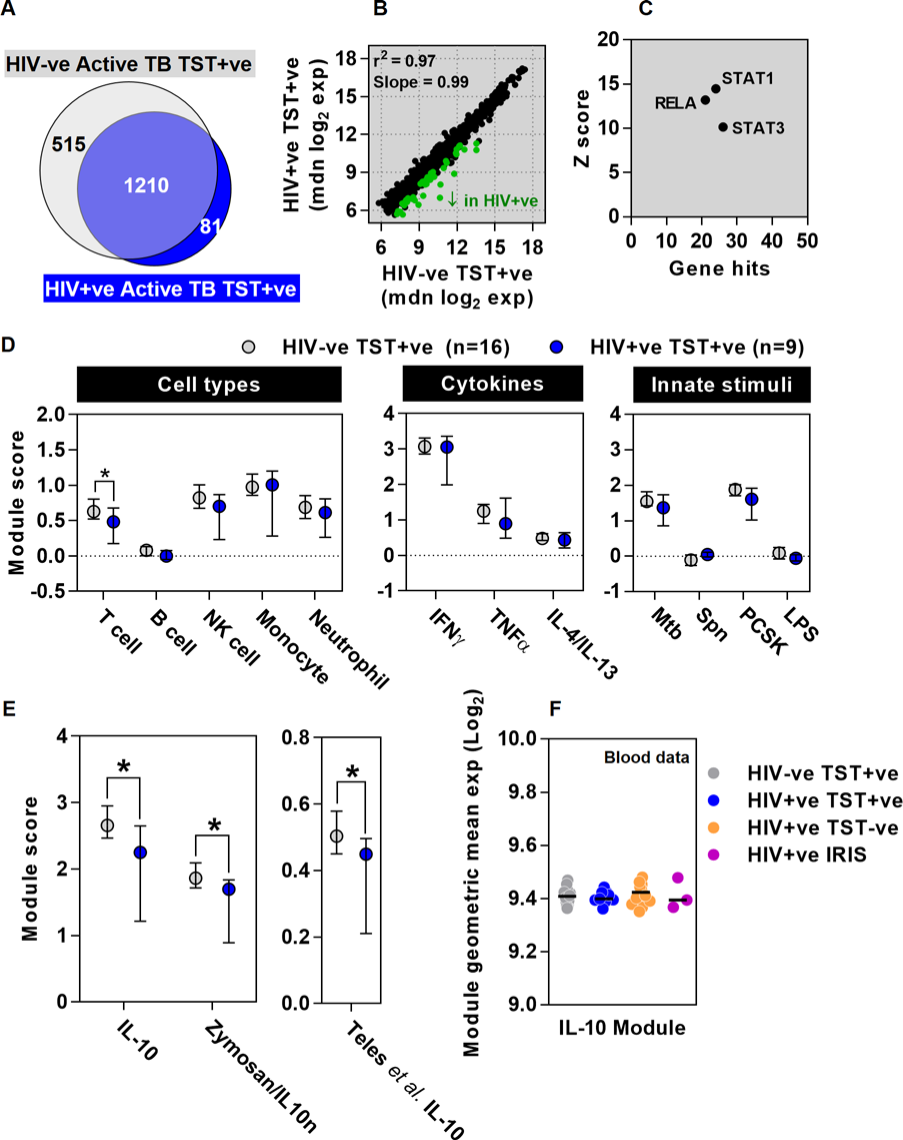
Attenuation of IL10 responses in HIV-1/TB co-infected patients with clinically positive TSTs. Comparison of increased transcript abundance in the TST of patients with active TB and clinically positive TSTs with (n = 9) and without (n = 16) HIV-1 infection, relative to the mean of saline controls (n = 8) is shown as a (a) Venn diagram, (b) scatter plot of median gene expression showing correlation (r^2^) and covariance (slope), highlighting the genes that show statistically significant decreased expression in green and (d) enrichment of cell-type and stimulus specific transcriptional modules. (c) TFBS enrichment analysis indicating the number of genes and statistical enrichment (Z score) associated with selected transcription factors is shown for genes that show lower transcript levels in HIV-1 infected patients. (e) Relative enrichment of two novel IL-10 inducible gene expression modules, and one previously published IL-10 module [47] in TSTs of HIV-1 infected and uninfected patients compared to saline controls. Data points (d-e) represent median ±IQR, *p<0.05 Mann-Whitney test). (f) IL10 module expression in the peripheral blood of these patient groups, showing data points for individual patients and median for each group.

The TST transcriptome in HIV-1 infected patients with positive TSTs showed marked overlap, correlation and covariance with that of HIV seronegative patients (Fig 3A and 3B). As might be expected from the effects of HIV-1 infection on Mtb-reactive CD4 T cell populations [14], modular analysis showed reduced abundance of T cell associated transcripts in the TST, but other selected cell type and pre-defined stimulus-specific modules were comparable in the two groups (Fig 3D). Comparison at the level of individual genes, identified significantly lower transcript abundance of a subset of genes in the TST of HIV-1 infected patients (Fig 3B). In order to evaluate the mechanism for the lower levels of these transcripts, we performed TFBS enrichment analysis on all genes significantly reduced in the TST signature of these HIV-infected patients (Fig 3C and S1 File). Enrichment of binding sites for STAT1 and NFκB RelA amongst the genes that showed diminished levels in HIV-1 infected patients suggested that IFN and TNFα or innate immune inducible genes, dependent on STAT1 and RelA respectively [40,41], might be attenuated in this group of HIV-1 infected patients. This hypothesis was not supported by modular analysis, which showed no difference in IFNγ, TNFα or innate immune stimulated gene expression modules (Fig 3D). However, we also found enrichment of STAT3 binding sites in the group of attenuated transcripts. STAT3 is the principal mediator of IL10 inducible transcriptional regulation [42,43], suggesting that IL10 activity may be attenuated in the TST within this group of HIV-1 infected patients.

IL10 upregulation in TB has been extensively described and experiments in mouse models have mostly focussed on its potential to compromise anti-mycobacterial host defences by regulating Th1 responses [44]. In contrast, recent data from non-human primate models suggest IL10 contributes to the optimal balance of host-protective immune responses within TB granulomas [12,45]. In addition, we reported in vitro data showing that HIV-1 infection of macrophages attenuates IL10 responses to co-infection with Mtb, leading to exaggerated inflammation [46]. Hence, increased risk of active TB in HIV-1 infected patients with preserved Th1 immunity, may principally arise from deficient IL10 immunoregulation. Therefore, we tested the hypothesis that this group of HIV-1 infected patients exhibit attenuated IL10 responses to the TST, in comparison to HIV seronegative patients, by using three independently derived modules for IL10 inducible genes. One previously published module was derived from IL10 stimulation of peripheral blood mononuclear cells [47] and two new modules we derived experimentally by stimulation of MDM with IL10 or by neutralising IL10 in zymosan stimulated MDM (S4 File and S9A–S9D Fig). In HIV seronegative patients with active TB, all three IL10 modules were enriched in the TST compared to control saline injection, confirming induction of IL10 activity in the host response to TST. Consistent with the hypothesis we derived from over representation of STAT3 TFBS amongst transcripts attenuated in HIV-1 infected patients, expression of each of the three IL10 modules was significantly less in HIV-1 infected patients with clinically positive TSTs (Fig 3E). These differences were not evident in whole blood transcriptional profiles from the same patients (Fig 3F).

### Preservation of type 1 IFN responses in HIV-1 infected patients with clinically negative TSTs

**Fig 4:**
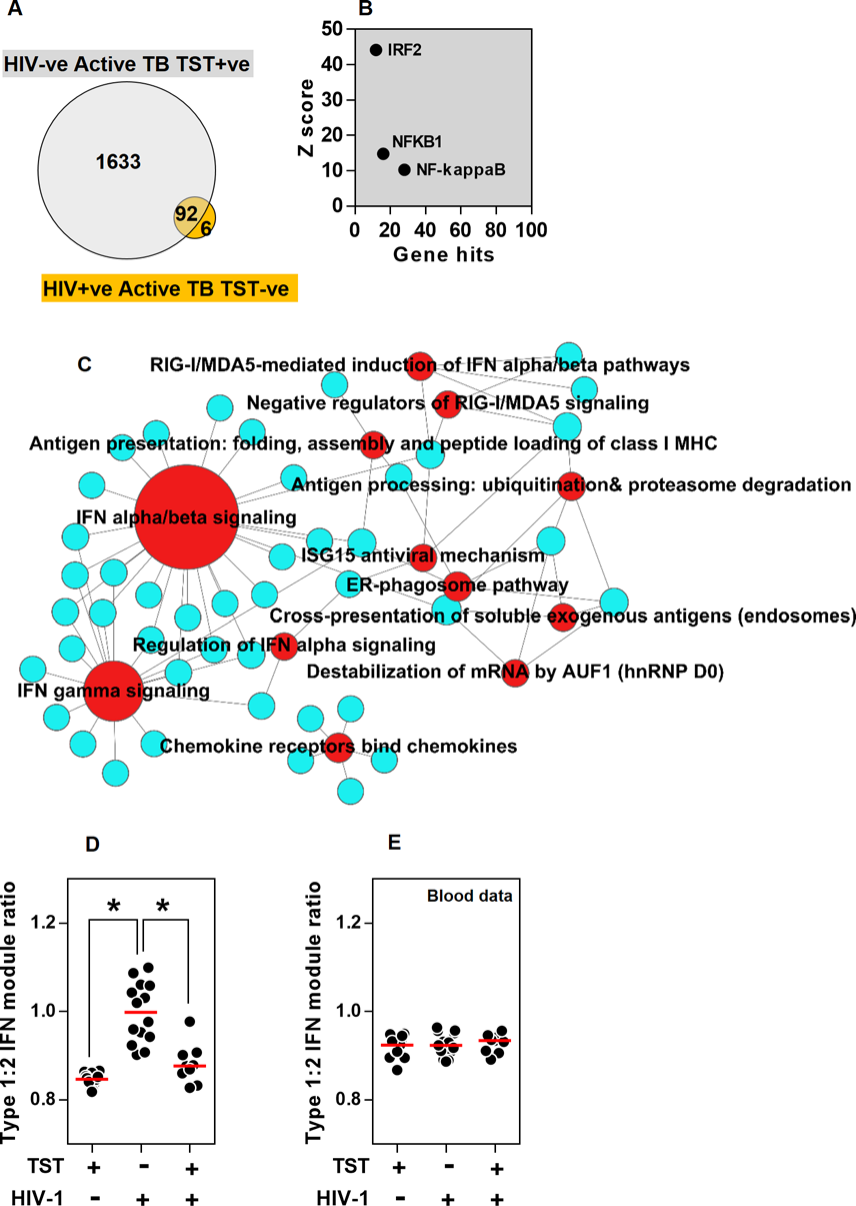
Preserved type 1 IFN responses uncoupled from other immune responses in HIV-1/TB co-infected patients with clinically negative TSTs. (a) In patients with active TB, the TST signature in HIV-1 negative patients with clinically positive TSTs (n = 16) and HIV-1 infected patients with clinically anergic TST (n = 14) are compared in a Venn diagram. For genes that show increased expression in this group of HIV-1 infected patients relative to the mean of saline controls (n = 8), (b) TFBS enrichment analysis is shown indicating the number of genes and statistical enrichment (Z score) associated for selected TFs, and associations with twelve most statistically enriched REACTOME functional pathways is represented in a network plot (c), in which the edges indicate associations between genes (blue nodes) and named pathways (red nodes), and the node size is proportional to the number of associations. Ratios of the median signal for stimulus specific type 1:2 IFN modules are shown within the (d) TST and (e) blood transcriptomes for different groups of patients with active TB as indicated, showing data points for individual patients and median for each group (*p<0.05 Mann-Whitney test).

Blood CD4 counts in TB/HIV-1 co-infected patients with clinically negative TSTs were significantly lower than co-infected patients with clinically positive TSTs, as previously reported [30] (Table 1 and S1 Fig). In keeping with the lack of significant clinical inflammation, their TST transcriptomes showed many fewer increases in transcripts compared to saline, than the TST transcriptome of HIV seronegative patients with active TB (Fig 4A) who had clinically evident responses (Table 1). Importantly TB/HIV-1 co-infected patients with clinically negative TSTs also had a substantially reduced TST transcriptome compared to healthy volunteers with clinically negative TSTs [29], who did have an immune response evident at the molecular level despite the absence of significant clinical induration (S10A Fig). Therefore, we concluded that TB/HIV-1 co-infected patients with clinically negative TSTs exhibit severe immunodeficiency affecting the broad range of innate and adaptive immune responses (S10B Fig). Nonetheless, changes in TST transcript abundance in 98 genes were evident in this group (Fig 4A). These genes showed most significant TFBS enrichment for IRF2 (Fig 4B and S1 File) thus implicating a role for IFN regulated pathways [48]. Pathway analysis of the 98 gene signature revealed that they were most strongly associated with type 1 IFN signalling, in contrast to the TST transcriptome of HIV seronegative patients with active TB, in whom more transcripts were associated with the type 2 IFN signalling pathway (Fig 4C and S2 File).

Recent evidence suggests that type 1 IFN responses may be detrimental to host defence against mycobacteria and that the ratio of type 1 versus type 2 IFN responses may contribute to clinical outcome [11,47]. There is considerable overlap between type 1 and type 2 IFN stimulated genes, therefore to validate our results, we derived exclusive type 1 (IFNβ) or type 2 IFN(γ) inducible gene modules by in vitro stimulation of MDM (S4 File and S9E and S9F Fig) and compared the ratio of type 1 to type 2 IFN stimulated genes in each study group. Consistent with the pathway analysis, our modular analysis showed significantly greater type 1:type 2 IFN activity in HIV-1 infected patients with negative TST, compared to those with positive TST and to HIV seronegative patients (Fig 4D). These differences were not reflected in the peripheral blood transcriptome of the same patients (Fig 4E). Of note, type 1 IFN has been reported to upregulate IL10 expression in human peripheral blood monocytes [47]. We therefore tested the hypothesis that increased type1:2 IFN module ratio within the TST may correlate with IL10 activity. In fact, by integrating data from HIV-1 negative and non-IRIS HIV-1 infected patients, we found significant inverse correlations between the ratio of type1:2 IFN activity and each of the three IL10 modules we have employed in the present study (S11A Fig). Interestingly, we also found no direct induction of IL10 upon stimulation of MDM with type 1 IFN, despite induction of other established IFN-inducible genes (S11B–S11D Fig).

### Exaggerated Th2 responses in HIV-1 patients with TB-IRIS

**Fig 5:**
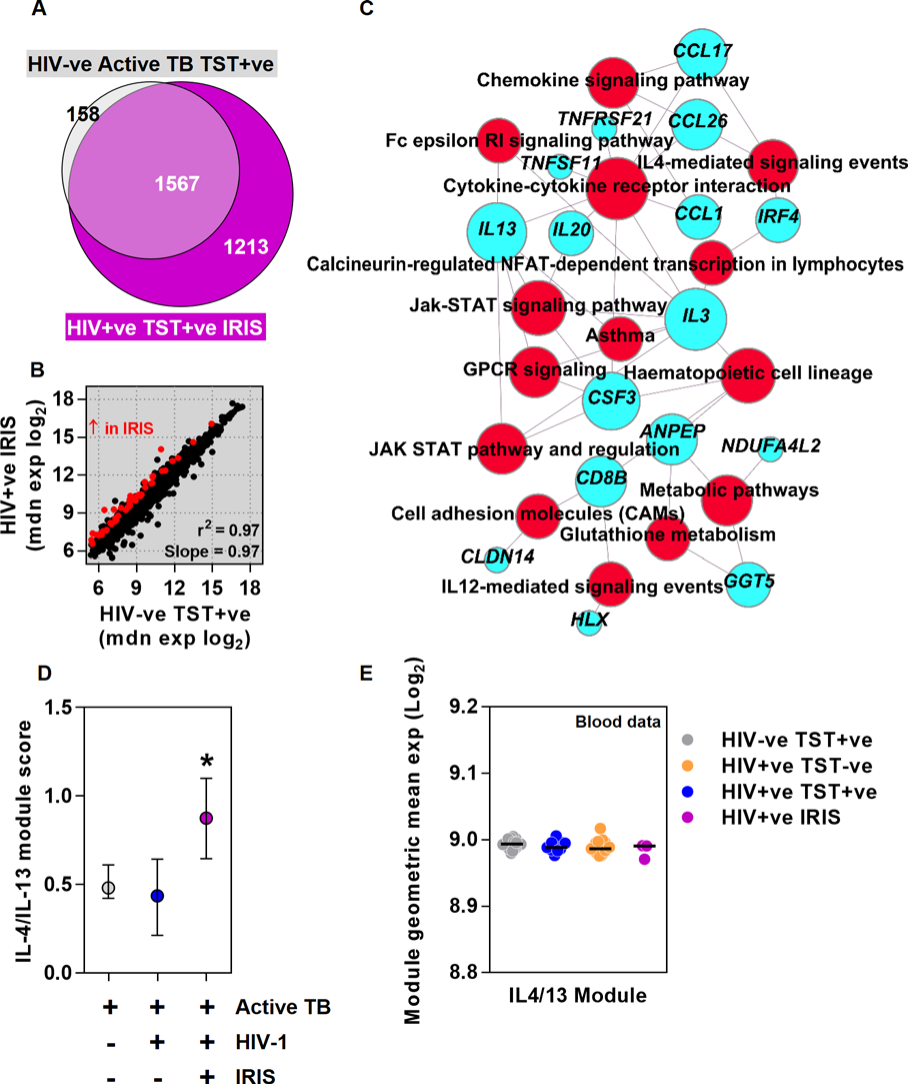
Exaggerated Th2 responses in HIV-1 infected patients with unmasking TB-IRIS. In patients with active TB, the TST signature in HIV-1 negative patients with clinically positive TSTs (n = 16) and HIV-1 infected patients with unmasking IRIS (n = 3), relative to the mean of saline controls (n = 8), are compared by (a) Venn diagram, and (b) scatter plot of median gene expression showing correlation (r^2^) and covariance (slope), highlighting the genes that show statistically significantly increased expression in red. (c) For genes that show increased expression in HIV-1 infected TB-IRIS patients, associations with statistically enriched functional pathways is represented in a network plot in which the edges indicate associations between genes (blue nodes) and named pathways (red nodes), and the node size is proportional to the number of associations. Relative enrichment of IL4/13 stimulated gene expression modules in (d) TST (median ±IQR), and (e) peripheral blood transcriptomes (data points for individual patients and median for each group) in the groups indicated (*p<0.05 Mann-Whitney test).

**Fig 6:**
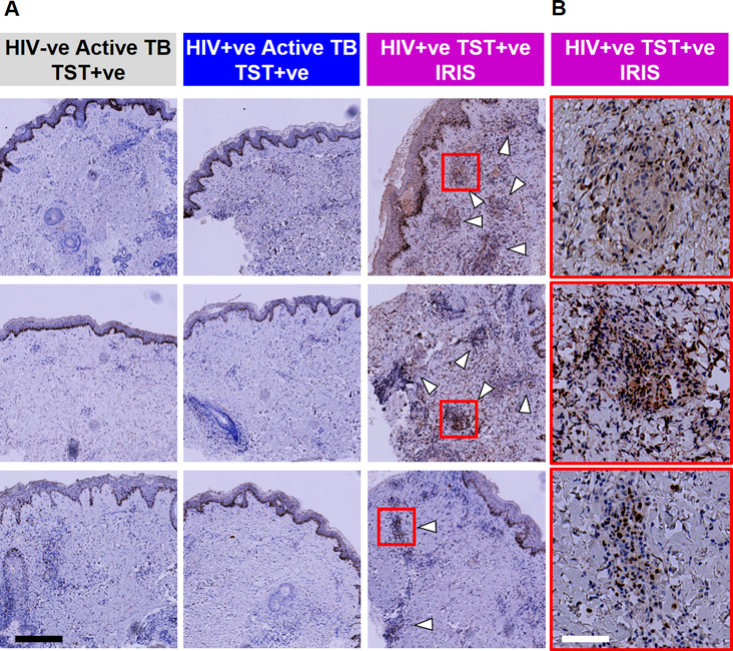
Increased immunostaining for IRF4 in HIV-1 infected patients with unmasking TB-IRIS. (a) Immunostaining of IRF4 in TST biopsies from three separate patients from each of the study groups indicated (white arrows indicate areas of positive IRF4 staining associated with inflammatory infiltrates). (b) Selected inflammatory cell infiltrates in TB-IRIS cases (indicated by red squares) are shown at higher magnification. Black scale bar = 400 μM and white scale bar = 80 μM.

ART substantially reduces the risk of incident TB disease associated with HIV-1 infection [49], but it can also exacerbate the immunopathology of TB and presentation of active TB after starting ART is sometimes attributed to unmasking immunopathology as a result of IRIS [50,51]. ART has already been associated with recovery of clinical responses to TST [52,53], but no specific immunological mechanism for IRIS has been established. In keeping with the case definition of IRIS [54], three HIV-1 infected patients who presented with TB two to eight weeks after starting ART had strong clinical TST responses (Table 1 and S1 Fig), which were also exaggerated at the transcriptional level when compared to HIV seronegative subjects (Fig 5A). 52 genes showed significantly increased expression in the TB-IRIS group compared with HIV-1 seronegative patients (Fig 5B). In pathway analysis, these transcripts were enriched for Th2 associated responses exemplified by genes linked to asthma and IL4-mediated signalling events (Fig 5C). This finding was confirmed by modular analysis, in which only the specific module for IL4 and IL13 stimulated genes in MDM, to model Th2 activity, was significantly enriched in the TST transcriptome of TB-IRIS patients compared to both HIV seronegative patients with active TB, and to HIV-1 infected patients with clinically positive TSTs presenting with active TB that was not temporally associated with recent initiation of ARVs (Fig 5D and S10C Fig). Further genome-wide comparisons of the TST transcriptomes of unmasking IRIS patients with those of non-IRIS TST positive HIV-1 infected patients also showed that unmasking IRIS was associated with an expanded TST transcriptome, with a subset of genes that show significantly higher transcript abundance enriched for Th2 associated pathways (S12 Fig). Once again, these transcriptional differences in the TST were not evident in peripheral blood (Fig 5E). We also sought to validate our observations in the TST transcriptome at the protein level by assessing IRF4, which is strongly implicated in induction of Th2 responses [55]. Increased transcriptional expression of IRF4 in the TSTs of unmasking TB-IRIS cases (Fig 5C) was mirrored by increased protein expression demonstrated by immunostaining of IRF4 (Fig 6).

## Discussion

Transcriptional profiling of the TST provides genome-wide assessments of in-situ human immune responses to a standardised challenge. Using this approach, we quantified Mtb specific innate responses, cellular recruitment and cytokine activity within the TST. We demonstrated that the same changes are evident in the transcriptome of human pulmonary TB granulomas, and were quantitatively correlated to the degree of immunopathology as measured by the MDH. Hence, the transcriptional profile of the TST provides an accurate model of the immune responses observed in TB lung granulomas, with better resolution than peripheral blood profiling. In order to undertake this analysis, we used the only five published transcriptional data sets from caseous pulmonary TB granuloma. More transcriptomic data comparing pathological lesions from the site of TB disease to normal tissue, and comparing TB granuloma at different stages of maturation, are necessary to consolidate our findings. In addition, we assessed the TST at 48 hours in line with clinical practice [28,56], but measurements at earlier and later time points may help build a dynamic molecular model of its initiation, amplification and resolution, each of which may contribute to differences in outcome.

In order to understand the impact of HIV-1 infection on immune responses to Mtb, we reasoned that TB/HIV-1 co-infected patients with preserved clinical TST responses, those with clinically anergic TST responses and patients presenting with unmasking TB-IRIS, will each reflect distinct mechanisms of immune dysfunction that increase risk of active TB. HIV-1 infected patients with preserved clinical TST responses and relatively well preserved circulating CD4 counts experience 3-5 fold greater risk of active TB [3]. Their TST transcriptional signature showed less enrichment of the T cell module, but IFNγ and TNFα activity, which are thought to mediate protective immunity to TB [31,57] were intact. Instead, we found evidence of deficient IL10 responses, which mirrored our previous report of deficient IL10 responses to Mtb by HIV-1 infected macrophages in vitro [46]. Given the immunoregulatory role of IL10 and recent focus on the importance of balanced immune responses in TB [10–12,45], we propose that HIV-1 infected patients with preserved Th1 immunity may incur higher risk of active TB as a result of deficient IL10 immunoregulation. Whether attenuation of TST IL10 responses in this group of HIV-1 infected patients is due to decreased IL10 production by virus infected cells or indirect effects of virus infection on the immune system, requires further investigation. Nonetheless our data suggest that stratification of patients by this phenotype or trials of immunoregulatory therapies merit investigation in this group of HIV-1 infected individuals.

TB/HIV-1 co-infected patients with clinically anergic TSTs had significantly depleted circulating CD4 counts and their TST transcriptome revealed almost complete immunological anergy with markedly reduced cellular recruitment, cytokine activity and innate immune responses. Importantly, this is in striking contrast to healthy HIV seronegative people with clinically negative TSTs, in whom the full repertoire of prototypic cell mediated immune responses are evident at the molecular level [29]. The degree of immunological anergy in HIV-1 infected patients with clinically negative TSTs is likely to underpin the 20-30 fold increased risk of TB and atypical mycobacterial infection associated with advanced HIV-1 disease [2,58]. It is interesting to speculate that immunodeficiency of this severity may also contribute to the relative failure of isoniazid preventative therapy in HIV-1 infected patients with clinically negative TSTs [59]. We infer that immune responses may therefore contribute to effective antimicrobial therapy, encouraging development of adjuvant host-directed therapies to improve TB treatment regimens. Remarkably, a type 1 IFN response to the TST was preserved in HIV-1 infected patients with clinically negative TSTs. This evidence extends human data for type 1 IFN responses to Mtb, and indicates that type 1 IFN responses may be uncoupled from the other innate immune responses that were severely attenuated in these patients. This finding is further supported by recent in vitro evidence for differential activation of IFN and inflammasome pathways [60]. In view of the data that suggest type 1 IFN responses may compromise host immunity to mycobacteria [11,47], these findings highlight the opportunity for specific therapeutic targeting of type 1 IFN activation pathways. Moreover, we hypothesise that increased proportions of type 1 to type 2 IFNs in this group of HIV-1 infected patients may further exacerbate their risk of active TB and contribute to the mechanisms of HIV immunodeficiency. Therefore, interest in targeting type 1 IFN activity to counteract chronic immune activation in HIV-1 infected patients [16,17], may also help to protect against TB. A mechanism by which increased ratios of type 1:2 IFN activity may compromise host protective immunity in TB is thought to be mediated by upregulation of immunosuppressive IL10 responses [47,61,11]. Of note, we did not find a positive correlation between type 1:2 IFN activity and IL10 activity in the TST. In addition, we found no direct type 1 IFN induction of IL10 expression in MDM, which has been previously reported in human monocytes [47]. Therefore, we conclude that this potential mechanism is unlikely to play a role in advanced HIV-1 infection.

HIV-1 infected patients presenting with TB two to eight weeks after starting ART, showed exaggerated transcriptional responses to the TST in keeping with the consensus case definitions for IRIS [54]. Both bioinformatic and independently derived gene module analysis provided entirely new insight into the immunopathogenesis of TB-IRIS, implicating a role for exaggerated Th2 polarised responses rather than increases in Th1 or innate immune responses. This Th2 bias was further confirmed by immunohistochemistry. This phenotype was statistically significant despite our very limited sample of three patients, but clearly merits further evaluation in future studies as it suggests that in the context of TB-IRIS at least, strategies targeting Th2 responses specifically should be considered for therapeutic intervention and may underpin the beneficial effects of glucocorticoid therapy in TB-IRIS [62,63].

Importantly, the effects of HIV-1 described above were specific to the site of immunological challenge and not evident in the steady state peripheral blood transcriptome of the same patients. Transcriptional profiling of the TST revealed differences in immune responses that were previously unrecognised by clinical assessments alone or previous laboratory investigations. Hence, we have identified novel and diverse molecular mechanisms by which human immune responses to Mtb are dysregulated in HIV-1 co-infected patients. Further validation of our observations in independent prospective cohorts is necessary. These data will inform rational development of host-directed adjuvant therapies to target specific immunological dysfunction in different patient groups.

## Materials and Methods

### Study approval

This study was approved by UK National Research Ethics Service (reference no: 11/LO/1045) and the University of Cape Town Human Research Ethics Committee (reference no: 580/2012).

### Study population

HIV-1 seropositive and seronegative adult patients (>16 years age) attending TB clinics in London and TB inpatient facilities and clinics in Cape Town who fulfilled inclusion/exclusion criteria (S1 Table) within one month of starting treatment for active tuberculosis (TB) were invited to participate. Written informed consent was obtained from all patients included in the study.

### Study schedule and sampling

On recruitment to the study, whole peripheral blood was collected in Tempus tubes for RNA (Life Technologies). In addition HIV-1 infected patients provided peripheral blood samples for CD4 T cell and HIV-1 viral load measurements by routine clinical services. Then patients received 0.1 mL intradermal injection of two units tuberculin (Serum Statens Institute) or saline in the volar aspect of one forearm, and this site was marked with indelible ink. At 48 hours, the clinical response at the injection site was evaluated by measurement of the maximum diameter of inflammatory induration and two 3 mm adjacent punch biopsies were obtained from marked TST or saline injection site as previously described [29]. Clinical induration >10 mm at the injection site was categorized as a positive response. The demographic, clinical and laboratory data for each study group is summarised in Table 1.

### Whole genome transcriptional profiling and analysis software

Total RNA from skin biopsy samples and MDM was purified as previously described [29]. Total RNA from Tempus tubes was purified using the Tempus Spin RNA Isolation Kit (Ambion; Life Technologies) and globin mRNA was eliminated using the GlobinClear kit (Life Technologies) according to the manufacturer’s instructions. RNA was subject to DNase treatment using a TURBO DNA-free kit (Ambion, Life Technologies) as per the manufacturer’s instructions to remove contaminating genomic DNA. Quality and concentration of all RNA samples were assessed using the Agilent Bioanalyzer. Total RNA was amplified, reverse transcribed into cDNA and then to cRNA and labelled with Cy5 or Cy3 using the Agilent Low RNA Input Linear Amplification Kit. Cy3 and Cy5 labelled samples were hybridized to Agilent 8x60k arrays as per manufacturer’s instructions. Array images were acquired with Agilent’s dual-laser microarray scanner G2565BA and signal data were collected with Agilent Feature Extraction software (v9.5.1). Median Cy3 and Cy5 signal intensity was Log transformed and normalized using LOESS local linear regression against the mean signal of all the samples using the R package agilp (http://www.bioconductor.org/packages/release/bioc/html/agilp.html). Principal component analysis (PCA) was performed using the prcomp function in R. Significant gene expression differences between data sets were identified using t-tests for parametric (normally distributed) data and Mann Whitney tests for non-parametric data in MultiExperiment Viewer v4.9 (http://www.tm4.org/mev.html) and a filter for >two-fold difference in mean or median normalised expression values. Pathway analysis was performed in innateDB [64] and transcription factor binding site enrichment analysis was performed using the human single site analysis function in oPossum [65] with default parameters in each application. Network graphics of gene and pathway association were generated using Gephi (http://gephi.github.io/). All microarray data used in this study are available in ArrayExpress (https://www.ebi.ac.uk/arrayexpress/) under the accession numbers provided in S2 Table.

### Identification of the TST gene expression signature

The clinical response to saline injection in HIV-1 infected and uninfected patients in this study was comparable (Table 1 and S2A and S2B Fig). Likewise, PCA of all the de novo data from HIV-1 infected and uninfected patients with active TB confirmed that genome-wide data from all subjects who received saline injection clustered together (S2C and S2D Fig). Specific comparison of data from HIV-1 infected and uninfected patients receiving saline showed significant differences (Mann Whitney test, p<0.05, fold-difference>2) in only 12 genes (S2E Fig), which were not significantly associated with any enrichment in pathway, gene ontology or transcription factor binding site enrichment analyses. Therefore, we concluded that HIV-1 infection did not significantly confound the response to saline and we pooled these data for further analysis. The TST gene signature in each group of patients represents RefSeq [66] annotated genes that showed significantly increased expression levels (Mann Whitney test, p<0.05, fold-difference>2) in skin biopsies from individual patients who received tuberculin were compared to that of all subjects who received control saline injections.

### Derivation of cell type specific gene modules

A gene expression matrix of purified cells was derived from the processed dataset E-GEOD-22886 available on ArrayExpress repository. The extracted data were not processed further aside from adding gene symbol annotations to probe names. Cell type specific modules were generated by identifying three to five gene probes corresponding to validated markers that identify each cell type of interest (S3 File). The markers were each used to identify co-correlated genes amongst all other gene probes in the expression data matrix. The top 1% of probes that were most co-correlated with the expression of each marker were identified using the Pearson correlation coefficient function in R, and cell type specific modules were then derived from the highly co-correlated probes that were common to all markers for each cell type (S3 File). The specificity of these modules was validated by comparison of the geometric mean expression level for each module within genome-wide data from each cell type in E-GEOD-22886 (from where the modules were derived) and also in E-GEOD-28490, an independent dataset of purified cell types (S4 and S5A and S5B Figs). Furthermore, the sensitivity of cell type specific modules to detect changes for specific cell populations in tissue specimens was evaluated in published data sets which described changes to cell composition confirmed by histological assessments (S5C–S5G Fig).

### Derivation of stimulus specific gene modules

MDM were generated as previously described [46]. Stimulus specific gene modules were derived from transcripts that were upregulated in MDM stimulated with selected cytokines, TLR ligands (LPS for TLR4 and Pam_2_CSK4 for TLR2), live *Mycobacterium tuberculosis* or *Streptococcus pneumoniae*. For cytokine-inducible gene expression modules, transcriptional profiling of human MDM was performed as described above after stimulation with recombinant human cytokines (S4 File). Significant transcriptional upregulation in comparison to unstimulated MDMs was identified by t-test with Welch’s approximation (p<0.05) and a ≥four-fold change threshold. The resulting gene lists were then compared and any which were upregulated ≥two-fold by more than one stimulus were excluded to create stimulus specific modules (S4 File). The specificity of each module was assessed by comparison of fold-changes in the geometric mean expression of genes in each module within MDM stimulated with each combination of stimuli compared to unstimulated MDM (S6A Fig). The same process was used to derive stimulus specific modules to distinguish between various innate immune stimuli in MDM (S4 File and S6B Fig).

Two IL10 specific modules were also derived by identifying genes that were upregulated in response to recombinant human IL10 stimulation of MDM (10 ng/mL for 24 hours) or by identifying gene expression changes attributable to an endogenous IL10 response in MDM following zymosan stimulation [67]. This was performed by identifying attenuated gene expression when using neutralising antibodies to IL10 and IL10 receptor as previously described [68], during 24 hour stimulation with zymosan (0.4 mg/mL). In the analysis of these data, statistical testing as described above was combined with a ≥two-fold change filter as too few genes were up-regulated ≥four-fold by IL10. Comparison of this gene list with the directly-induced IL-10 module revealed some overlap (S9A Fig) and both modules showed significant TFBS enrichment for STAT3 (S9C and S9D Fig), providing independent supporting evidence for generating valid IL10 associated gene expression modules using this approach. Comparison of these gene lists demonstrated minimal overlap and good functional specificity compared to other cytokine stimulation (S9B Fig). To complement these two IL10 inducible gene expression modules, we adopted a third module used to identify IL10 activity derived by 24 hour IL10 stimulation of PBMC in a previously published study [47].

Two further modules were derived to distinguish type 1 or type 2 IFN inducible gene expression using transcriptional data for MDM stimulated for 4 hours with 10 ng/mL of either type 1 (IFN β), or type 2 IFN (IFNγ). As expected, statistically significant upregulated gene expression (>2-fold) showed considerable overlap (S9E Fig), but stimulus-specific modules, derived as described above using a >4-fold threshold and exclusion of those upregulated by >2-fold by the other stimulus showed good specificity (S9F Fig).

### Identification of a an active TB gene signature in peripheral blood

The peripheral blood active TB gene signature in this study represents RefSeq annotated genes that showed significantly increased expression levels (Mann Whitney test, p<0.05, fold-difference>2) in peripheral blood transcriptomes of samples obtained prior to TST in HIV seronegative patients with active TB was compared to samples from HIV seronegative healthy volunteers Table 1.

### Quantification of module expression in TST, blood and lung samples

Module scores were derived by calculating log ratio of the geometric mean of expression data for each module within individual TST biopsies compared to pooled data from saline controls. For peripheral blood and lung transcriptional profiles, the relative enrichment of each module was assessed by presenting the geometric mean expression of the module gene list in each individual Log_2_ transformed expression profile.

### Quantitation of molecular distance to health in pulmonary active TB granulomatous lesions

Published gene expression data derived from human granulomatous lesions in pulmonary TB and healthy human lung tissue [35] was used. The molecular distance to health was derived as previously described [13] by calculating the sum of standard deviations (>2 SD) for data from each granulomatous lesion compared to the mean of data from healthy lung tissue.

### PCR and ELISA quantitation of MDM responses to type 1 IFN stimulation

Total RNA and culture supernatants were collected from MDM cultures stimulated with IFNβ, IFNγ or zymosan (S4 File). RNA was used to synthesise first strand cDNA using the qScript cDNA Supermix kit (Quanta BioSciences) and quantitative (q)PCR of IL10, IFI16 and GAPDH was performed using TaqMan inventoried assays (Applied Biosystems) using according to the manufacturer’s instructions. IL10 protein was quantified in culture supernatants by ELISA (eBioscience) according to the manufacturer’s instructions.

### Histology and immunohistochemistry of skin biopsy specimens

Punch skin biopsies for histological analysis were collected into 4% neutral buffered formalin (NBF; LabSource) for fixation, then embedded in paraffin, sectioned and mounted on slides, for staining with haemotoxylin and eosin or immunostaining with Mouse anti-human IRF4 (clone MUM1P from DAKO). 3μm paraffin sections underwent automated dewaxing and antigen retrieval using Leica Bond ER2 (pH9) at 100°C for 20 minutes, followed by peroxide blocking for 5 minutes at room temperature. Sections were then incubated sequentially with the primary antibody for 15 minutes, rabbit-anti-mouse secondary antibody and anti-rabbit poly-HRP followed by DAB (Bond Refine detection kit) and 0.5% copper sulfate. Leica Bond Wash and demineralised water were used for washing steps between reagent steps. Digital images were acquired with an AxioScan Z1 slide scanner (Zeiss) and presented without any subsequent processing. All histological grading was performed by a histopathologist blinded to concomitant clinical information. Histological scoring of inflammation was performed on a 0–3 scale, where 0 represented no evidence of inflammation and 3 was the most inflammation within the spectrum of samples.

## Supporting Information

S1 Fig Clinical and histological scoring of TSTs. Comparison of (A) clinical TST responses and histological scores, and (B) CD4 counts in the different study groups indicated and relationship between CD4 counts and TST induration (data points represent individual subjects and median of each group, *p<0.05 Mann-Whitney test). (C) Representative images of histology associated with each inflammatory score amongst the different study groups (arrows indicate areas with inflammatory infiltrates). Grey panels represent classifications for which there were no patients. Scale bar = 400 μM.
(TIF)

S2 Fig Histological and transcriptional analysis at the site of saline injections. (A) Representative histology and (B) histological scoring from biopsies of saline injection sites. (C) Principle component analysis (PCA) of the transcriptome of saline injection sites in HIV-1 infected and uninfected subjects compared to all TST transcriptomes. (D) Hierarchical clustering of the transcriptome of saline injection sites in HIV-1 infected and uninfected subjects. (E) Gene expression heat map of all significantly different transcripts in saline injection sites in HIV-1 infected and uninfected subjects.
(TIF)

S3 Fig Principle component analysis of TST transcriptomes in HIV-1 negative patients with active TB grouped by duration of TB treatment before TST or by site of TB disease. PCA of significantly enriched transcripts in the TST biopsies of HIV-1 negative people with active TB, showing the first two components (with the proportion of variance across all samples represented by each component) and grouping the patients by duration of TB treatment at the time of TST (A), or by the site of TB disease (B). Given that these groups do not cluster separately, we infer from this analysis that neither time on treatment or site of disease cause major confounding of the transcriptional response to the TST.
(TIF)

S4 Fig Schematic analysis flow chart for discovery and validation of cell type specific gene expression modules. Cell type specific modules were generated by identifying top 1% of genes in whole genome transcriptional data (E-GEOD-22886) which show co-correlation with all of three to five gene probes corresponding to validated cell specific markers. The modules were then validated by testing cell-type specific enrichment in the discovery data set, an independent data set of purified cell types (E-GEOD-28490) and multiple tissue data sets (accession numbers shown) in which differential cellular composition has been evaluated by an independent method.
(TIF)

S5 Fig Validation of cell type specific gene expression modules. (A-B) Relative expression levels of cell type specific modules within transcriptional data from different cell types. (C-G) Geometric mean expression of each of the cell-type specific modules is shown in the specific transcriptional data sets indicated (data accession numbers and detailed in S2 Table). Module expression data is consistent with histological evidence for enrichment of B cells in interstitial pulmonary fibrosis (IPF) samples compared to normal lung (C), enrichment of T cells at the site of intradermal IFNγ injection compared to placebo (D), depletion of NK cells following treatment with Asoprisinil compared to placebo (E), enrichment of monocyte derived cells in mediastinal lymphnodes of patients with sarcoidosis compared to cancer (F) and enrichment of neutrophils in erythema nodosum leprosum (ENL) compared to lepromatous leprosy skin lesions (G).
(TIF)

S6 Fig Validation of stimulus specific gene expression modules. Fold change in geometric mean of each module in cytokine (A) or innate immune stimulus specific modules (B) are compared within transcriptional data from MDM stimulated with each stimulus, relative to unstimulated MDM. (LPS = lipopolysaccharide, PCSK = Pam_2_CSK4, Mtb = *Mycobacterium tuberculosis*, Spn = *Streptococcus pneumoniae*).
(TIF)

S7 Fig Modular analysis of TST transcriptomes in HIV-1 negative patients with active TB grouped by duration of TB treatment before TST or by site of TB disease. Cell type and stimulus specific module scores in the TST transcriptomes of HIV-1 negative people with active TB grouped by duration of TB treatment at time of TST challenge (A) or by site of TB disease (B). Module scores represent log ratio of geometric mean of gene expression for each module within TST compared to saline biopsies, showing data points for each individual case with median and range.
(TIF)

S8 Fig Comparison of TST and peripheral blood transcriptional changes in HIV-1 negative patients with active TB. (A) In HIV-1 seronegative patients with active TB, an equivalent number (top 1000) genes that showed statistically significant increased transcript abundance in peripheral blood samples relative to peripheral blood of healthy volunteers and increased transcript abundance within their TST transcriptomes relative to saline controls are compared in a scatter plot. (B) Gene expression that showed increased levels in one or both data sets (colour coded as indicated) were subjected to REACTOME pathway analysis to show statistically enriched associations.
(TIF)

S9 Fig IL10, type I and type II IFN-inducible gene expression modules. (A) Genes upregulated (>2-fold) by IL10 or downregulated (>2-fold) by neutralising IL10 activity after zymosan stimulation (Zymosan & IL10n) were compared in a Venn diagram. (B) Relative increase in geometric mean expression level of genes within cytokine specific modules are compared within transcriptional data from MDM stimulated with each stimulus, relative to unstimulated MDM. TFBS enrichment analysis is shown, indicating the number of genes and statistical enrichment (Z score) associated with the indicated TFs, for the IL-10 module (C) and the zymosan/IL10n module (D). Genes upregulated (>2-fold) by type 1 (IFNβ) or type 2 (IFNγ) stimulation of MDM were compared in a Venn diagram (E). Relative increase in geometric mean expression level of genes within cytokine specific modules are compared within transcriptional data from MDM stimulated with each stimulus, relative to unstimulated MDM (F).
(TIF)

S10 Fig Venn diagram and modular comparisons of TST transcriptomes in selected study groups. Comparison of the TST transcriptional signature of healthy HIV-1 negative subjects with clinically negative TST responses, HIV negative subjects with active TB and clinically positive TST responses and HIV-1 infected subjects with active TB and clinically negative TSTs, relative to saline controls is shown in (A) a Venn diagram, and for (B) cell-type and stimulus specific transcriptional modules. Data points represent median ±IQR. Single asterisk indicates statistically significant difference to HIV-1 negative patients with active TB. Double asterix indicates statistically significant difference to both groups of HIV-1 negative cases. (C) Cell-type and stimulus specific transcriptional modules in the TST transcriptome of HIV-1 seronegative patients with active TB and HIV-1 infected patients with TB-IRIS are compared relative to data from saline controls. Data points represent median ±IQR, *p<0.05 Mann Whitney test.
(TIF)

S11 Fig Correlation of type 1:2 IFN modules with IL10 modules and IL10 expression in type 1 IFN-stimulated MDM. (A) Scatter plot of type 1:2 IFN module score ratios and IL10 module score for three separate IL10 modules within the TST transcriptome of all non-IRIS HIV+ve patients with active TB. Module scores represent log ratio of geometric mean of gene expression for each module within TST compared to saline biopsies. Correlation coefficient (r^2^) and p-value are indicated for each regression line. (B) Quantitation of IL10 expression in MDM cultures ±stimuli indicated using qPCR or (C) ELISA of culture supernatants. (D) qPCR quantitation of IFI16 in MDM cultures ±stimuli indicated. All qPCR data normalised to GAPDH. (Bars indicate mean ±SEM, n = 4, *p<0.05 unpaired t-test).
(TIF)

S12 Fig Comparison of TST transcriptomes in non-IRIS HIV-1 infected study groups with clinically positive and negative TSTs. The TST signature in HIV+ve/TST+ve unmasking TB-IRIS (n = 3) and non-IRIS cases (n = 9), relative to the mean of saline controls (n = 8), are compared by (A) Venn diagram, and (B) scatter plot of median gene expression showing correlation (r^2^) and covariance (slope), highlighting the genes that show statistically significant expression in red, and corrected p value for statistically enriched functional pathways in these genes (C). Red bars indicate pathways associated with Th2 Tcell responses. Dotted line indicates–log_10_ p value of 1.3 equivalent to p<0.05.
(TIF)

S1 Table Inclusion and exclusion criteria and description of study groups. (DOCX)

S2 Table Table of accession numbers for all array data within ArrayExpress. (DOCX)

S1 File Transcriptional factor binding site enrichment analysis. (XLSX)

S2 File Genes associated with enriched pathways in Figs 1D, 4C and 5C. (XLSX)

S3 File Cell type specific gene expression modules. (XLSX)

S4 File Stimulus specific gene expression modules. (XLSX)
