# Supplementary figures and images for: In Vivo Molecular Dissection of the Effects of HIV-1 in Active Tuberculosis
Source: PLoS Pathog. 2016 Mar 17;12(3):e1005469. doi: 10.1371/journal.ppat.1005469 (PMC4795555; doi:10.1371/journal.ppat.1005469)

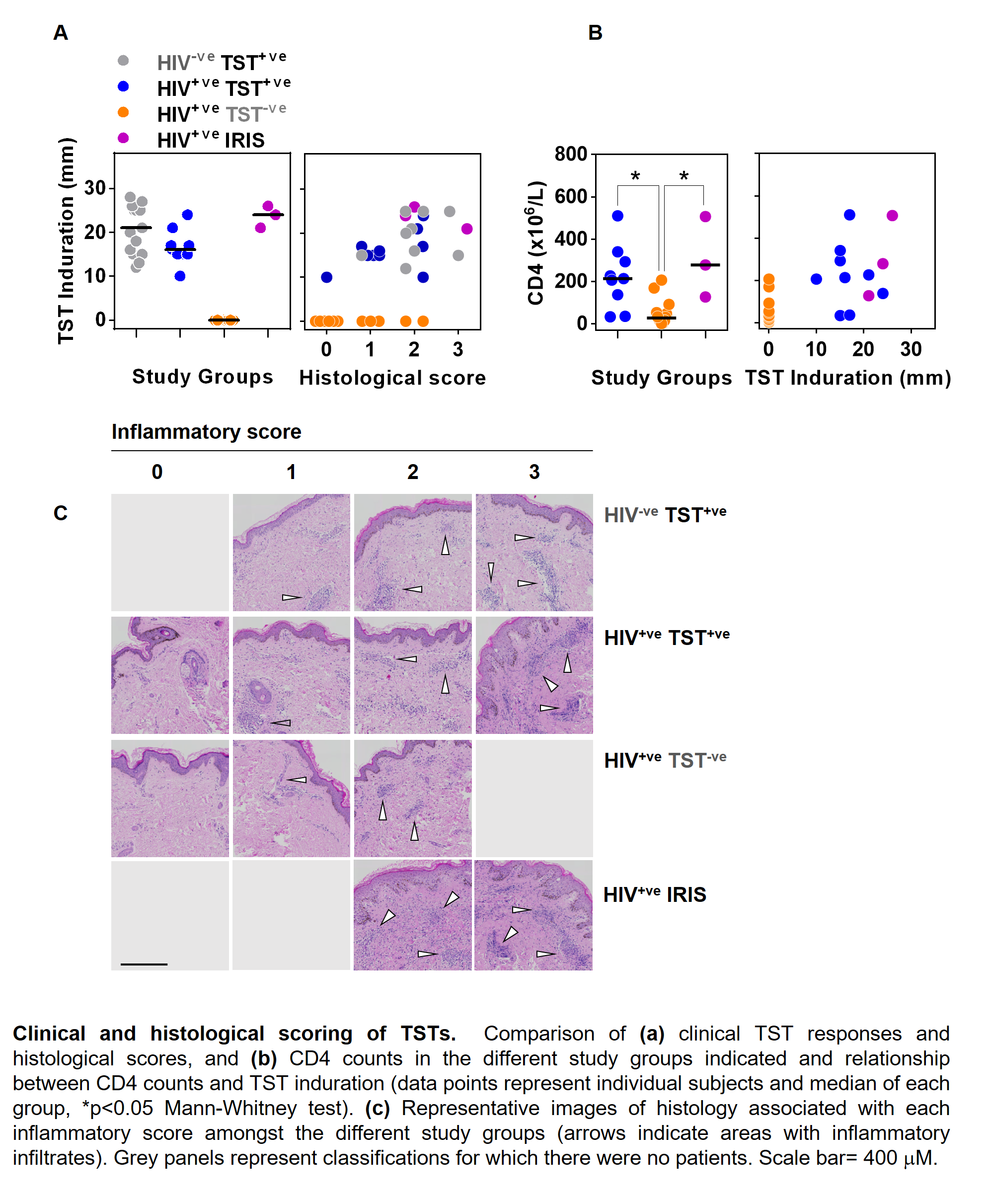

Supplement: S1 Fig — Comparison of (A) clinical TST responses and histological scores, and (B) CD4 counts in the different study groups indicated and relationship between CD4 counts and TST induration (data points represent individual subjects and median of each group, *p<0.05 Mann-Whitney test). (C) Representative images of histology associated with each inflammatory score amongst the different study groups (arrows indicate areas with inflammatory infiltrates). Grey panels represent classifications for which there were no patients. Scale bar = 400 μM. (TIF) [file ppat.1005469.s001.tif]

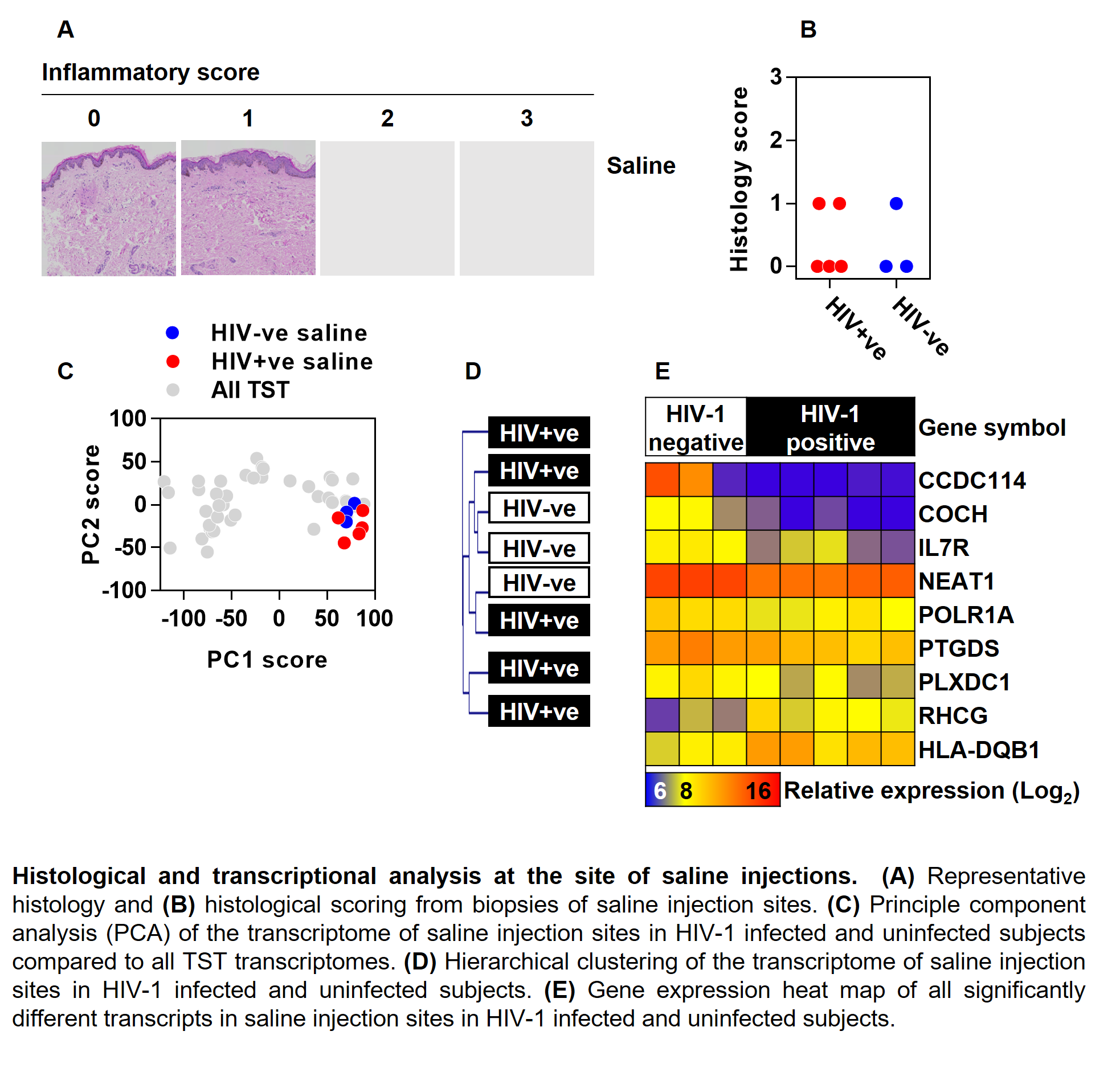

Supplement: S2 Fig — (A) Representative histology and (B) histological scoring from biopsies of saline injection sites. (C) Principle component analysis (PCA) of the transcriptome of saline injection sites in HIV-1 infected and uninfected subjects compared to all TST transcriptomes. (D) Hierarchical clustering of the transcriptome of saline injection sites in HIV-1 infected and uninfected subjects. (E) Gene expression heat map of all significantly different transcripts in saline injection sites in HIV-1 infected and uninfected subjects. (TIF) [file ppat.1005469.s002.tif]

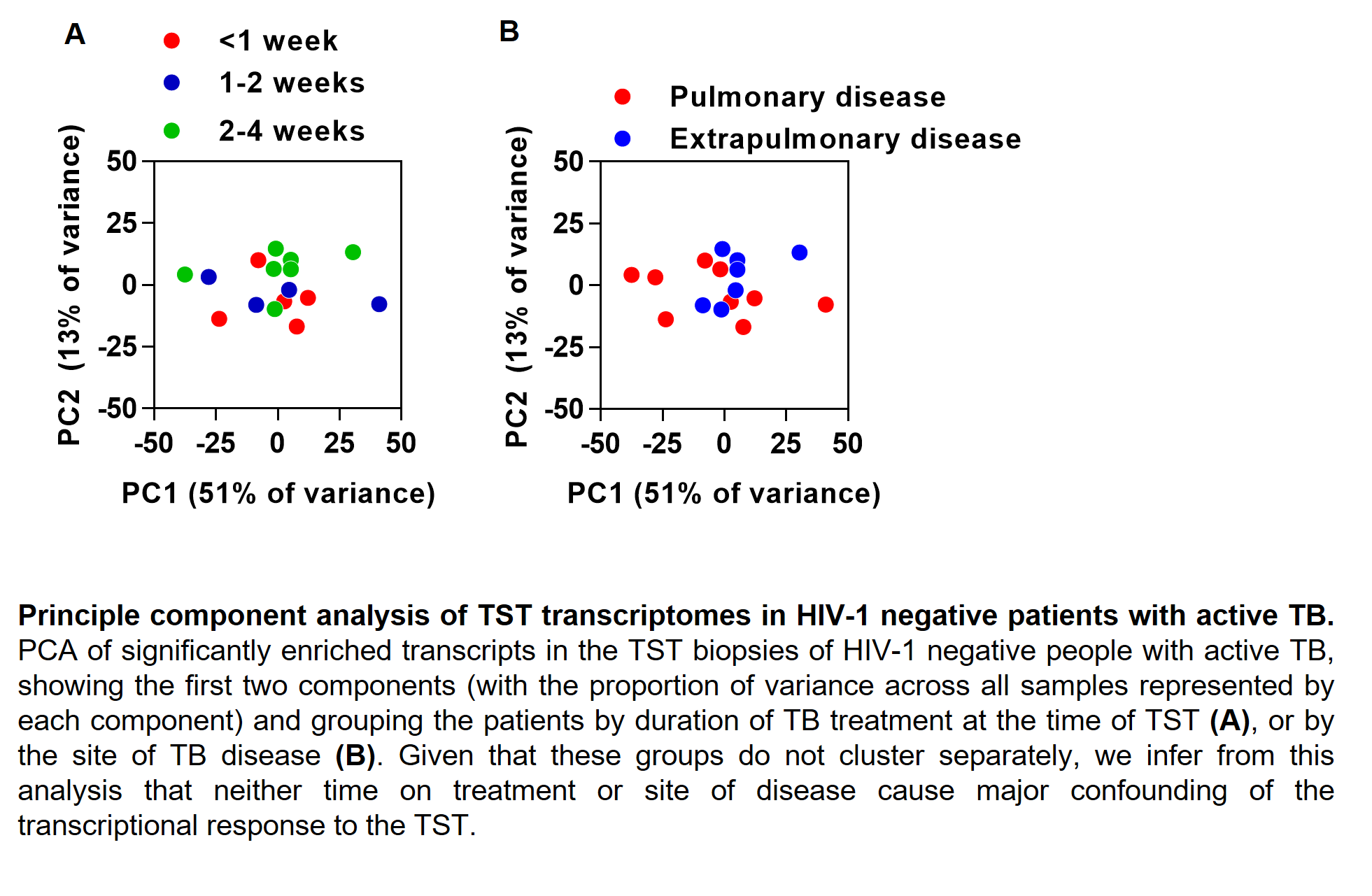

Supplement: S3 Fig — PCA of significantly enriched transcripts in the TST biopsies of HIV-1 negative people with active TB, showing the first two components (with the proportion of variance across all samples represented by each component) and grouping the patients by duration of TB treatment at the time of TST (A), or by the site of TB disease (B). Given that these groups do not cluster separately, we infer from this analysis that neither time on treatment or site of disease cause major confounding of the transcriptional response to the TST. (TIF) [file ppat.1005469.s003.TIF]

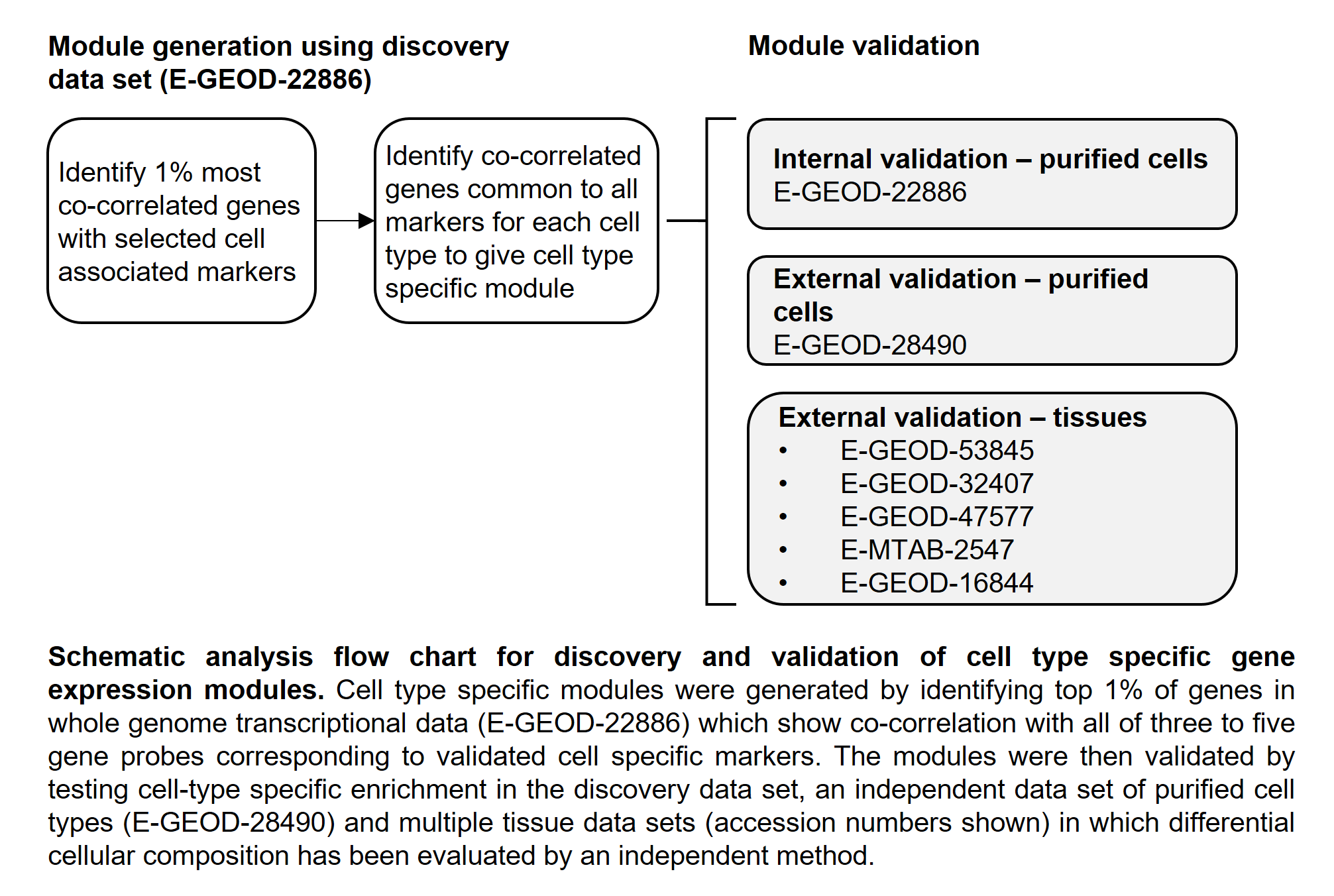

Supplement: S4 Fig — Cell type specific modules were generated by identifying top 1% of genes in whole genome transcriptional data (E-GEOD-22886) which show co-correlation with all of three to five gene probes corresponding to validated cell specific markers. The modules were then validated by testing cell-type specific enrichment in the discovery data set, an independent data set of purified cell types (E-GEOD-28490) and multiple tissue data sets (accession numbers shown) in which differential cellular composition has been evaluated by an independent method. (TIF) [file ppat.1005469.s004.tif]

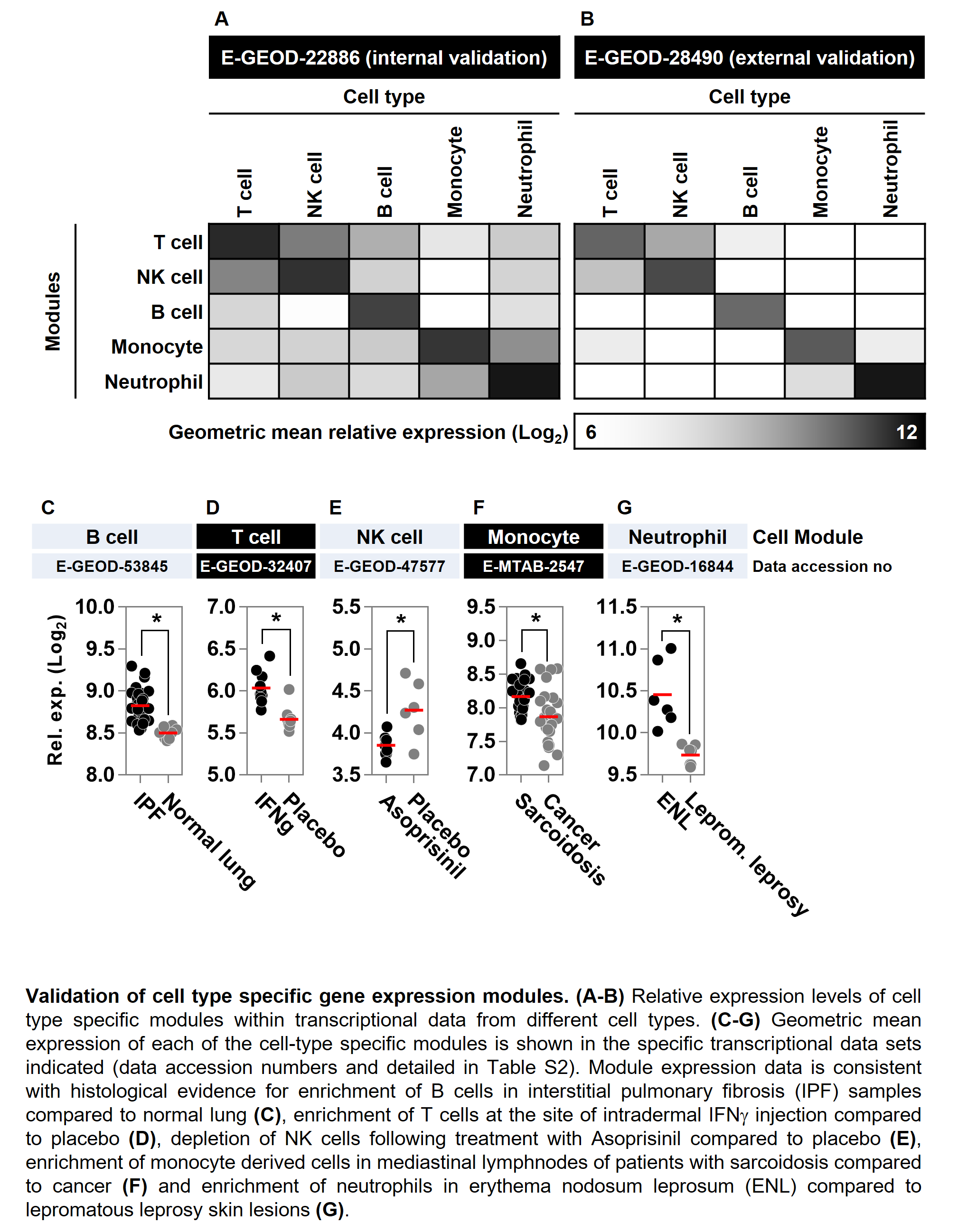

Supplement: S5 Fig — (A-B) Relative expression levels of cell type specific modules within transcriptional data from different cell types. (C-G) Geometric mean expression of each of the cell-type specific modules is shown in the specific transcriptional data sets indicated (data accession numbers and detailed in S2 Table). Module expression data is consistent with histological evidence for enrichment of B cells in interstitial pulmonary fibrosis (IPF) samples compared to normal lung (C), enrichment of T cells at the site of intradermal IFNγ injection compared to placebo (D), depletion of NK cells following treatment with Asoprisinil compared to placebo (E), enrichment of monocyte derived cells in mediastinal lymphnodes of patients with sarcoidosis compared to cancer (F) and enrichment of neutrophils in erythema nodosum leprosum (ENL) compared to lepromatous leprosy skin lesions (G). (TIF) [file ppat.1005469.s005.tif]

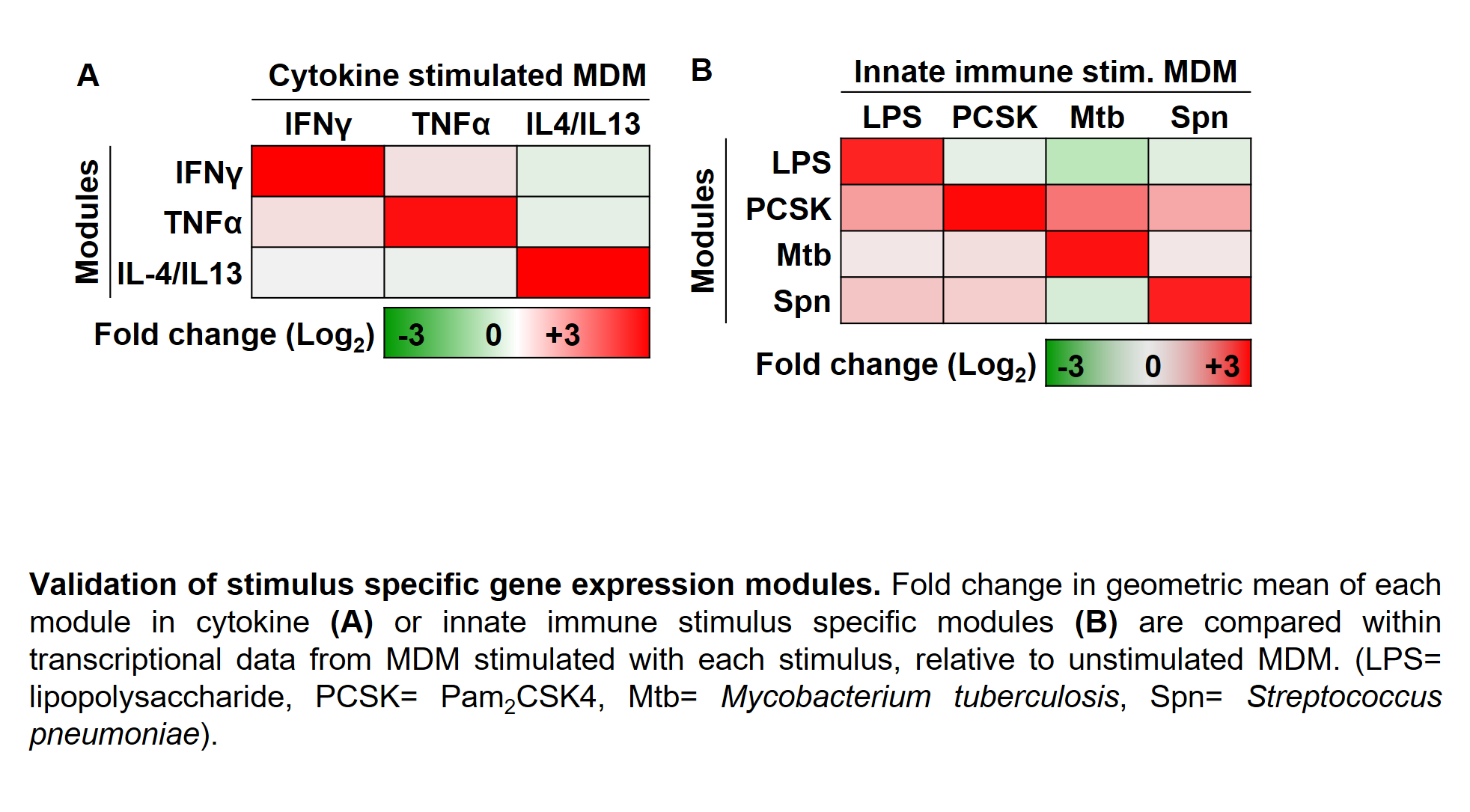

Supplement: S6 Fig — Fold change in geometric mean of each module in cytokine (A) or innate immune stimulus specific modules (B) are compared within transcriptional data from MDM stimulated with each stimulus, relative to unstimulated MDM. (LPS = lipopolysaccharide, PCSK = Pam2CSK4, Mtb = Mycobacterium tuberculosis, Spn = Streptococcus pneumoniae). (TIF) [file ppat.1005469.s006.tif]

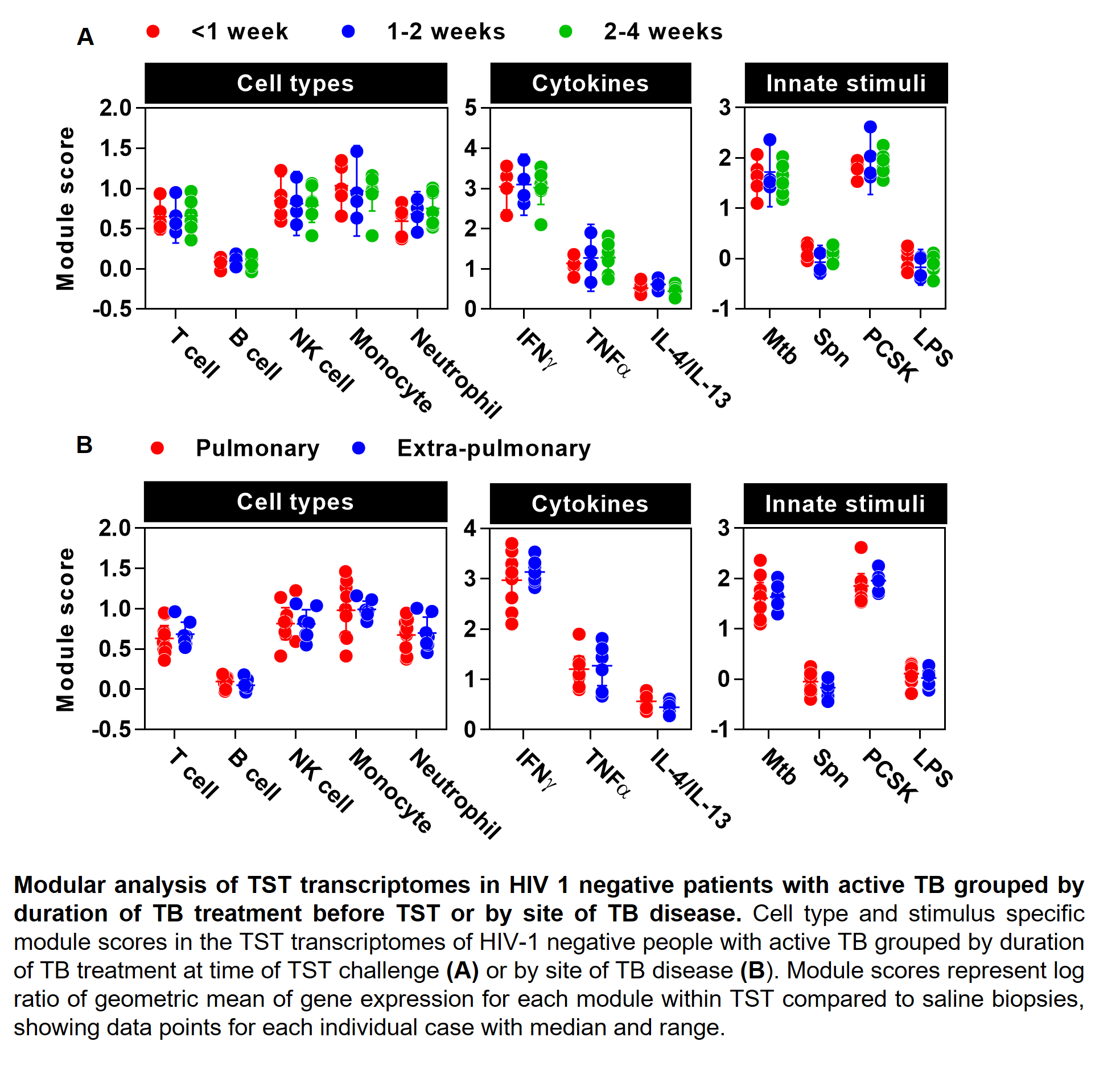

Supplement: S7 Fig — Cell type and stimulus specific module scores in the TST transcriptomes of HIV-1 negative people with active TB grouped by duration of TB treatment at time of TST challenge (A) or by site of TB disease (B). Module scores represent log ratio of geometric mean of gene expression for each module within TST compared to saline biopsies, showing data points for each individual case with median and range. (TIF) [file ppat.1005469.s007.tif]

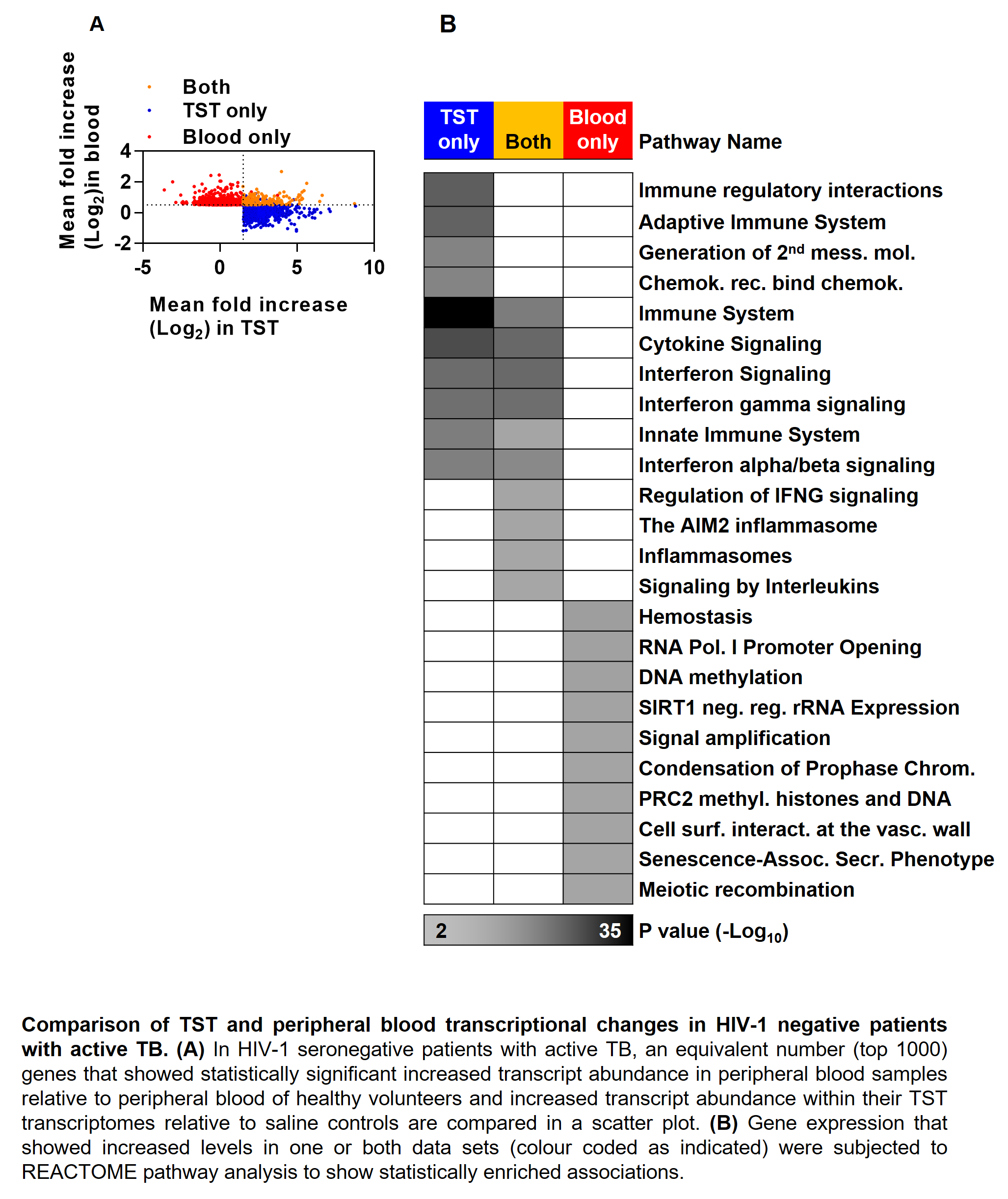

Supplement: S8 Fig — (A) In HIV-1 seronegative patients with active TB, an equivalent number (top 1000) genes that showed statistically significant increased transcript abundance in peripheral blood samples relative to peripheral blood of healthy volunteers and increased transcript abundance within their TST transcriptomes relative to saline controls are compared in a scatter plot. (B) Gene expression that showed increased levels in one or both data sets (colour coded as indicated) were subjected to REACTOME pathway analysis to show statistically enriched associations. (TIF) [file ppat.1005469.s008.tif]

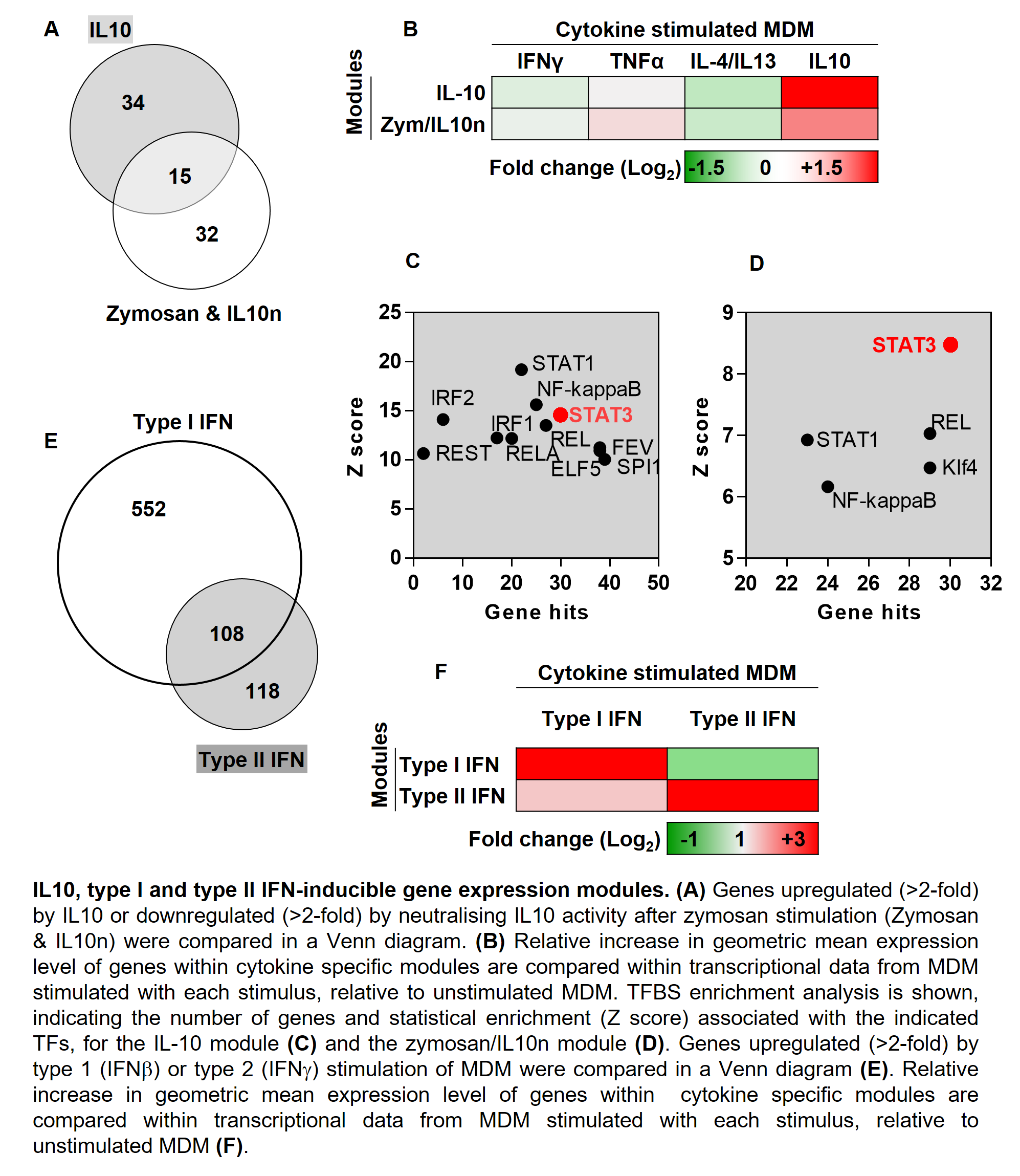

Supplement: S9 Fig — (A) Genes upregulated (>2-fold) by IL10 or downregulated (>2-fold) by neutralising IL10 activity after zymosan stimulation (Zymosan & IL10n) were compared in a Venn diagram. (B) Relative increase in geometric mean expression level of genes within cytokine specific modules are compared within transcriptional data from MDM stimulated with each stimulus, relative to unstimulated MDM. TFBS enrichment analysis is shown, indicating the number of genes and statistical enrichment (Z score) associated with the indicated TFs, for the IL-10 module (C) and the zymosan/IL10n module (D). Genes upregulated (>2-fold) by type 1 (IFNβ) or type 2 (IFNγ) stimulation of MDM were compared in a Venn diagram (E). Relative increase in geometric mean expression level of genes within cytokine specific modules are compared within transcriptional data from MDM stimulated with each stimulus, relative to unstimulated MDM (F). (TIF) [file ppat.1005469.s009.tif]

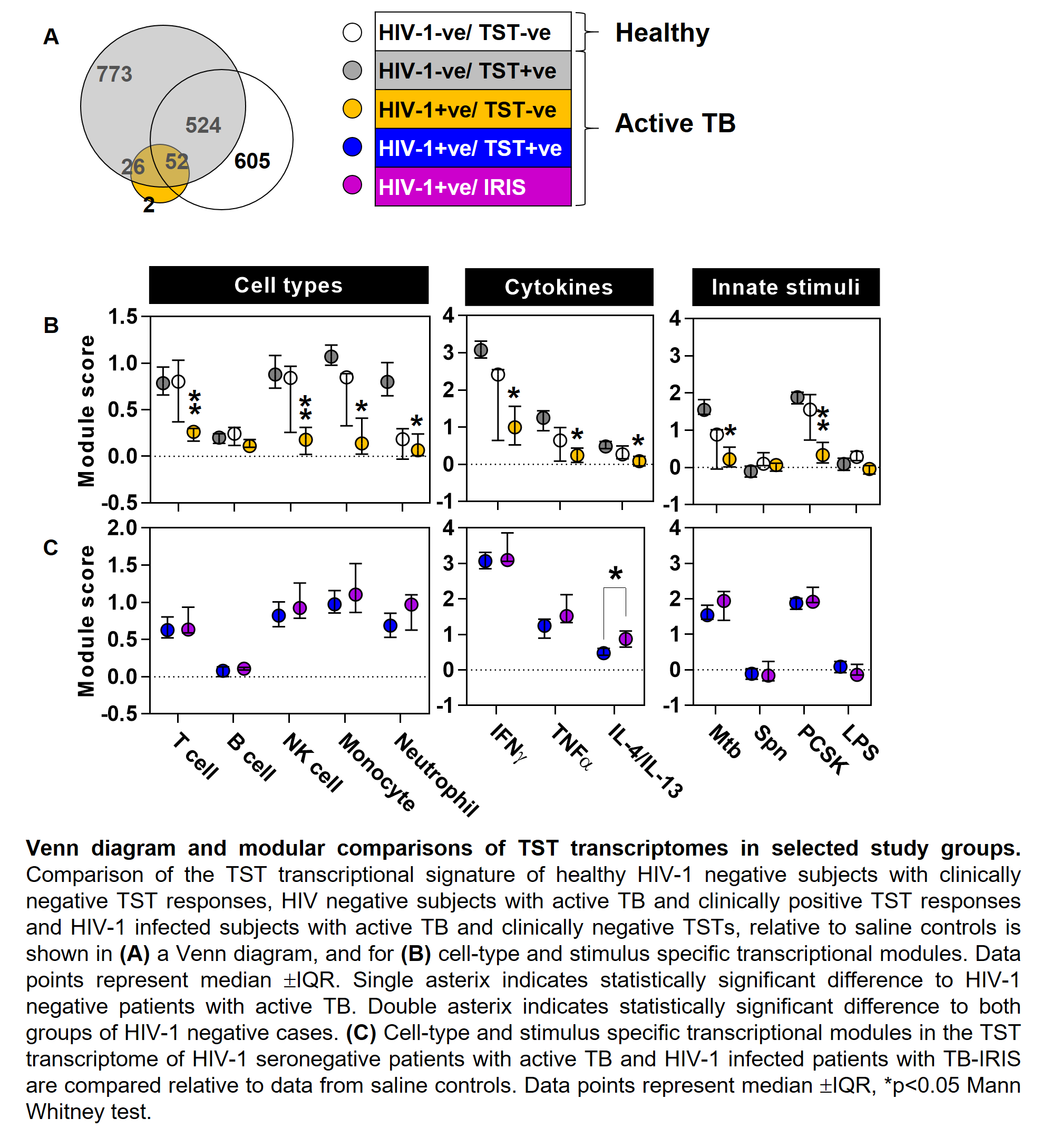

Supplement: S10 Fig — Comparison of the TST transcriptional signature of healthy HIV-1 negative subjects with clinically negative TST responses, HIV negative subjects with active TB and clinically positive TST responses and HIV-1 infected subjects with active TB and clinically negative TSTs, relative to saline controls is shown in (A) a Venn diagram, and for (B) cell-type and stimulus specific transcriptional modules. Data points represent median ±IQR. Single asterisk indicates statistically significant difference to HIV-1 negative patients with active TB. Double asterix indicates statistically significant difference to both groups of HIV-1 negative cases. (C) Cell-type and stimulus specific transcriptional modules in the TST transcriptome of HIV-1 seronegative patients with active TB and HIV-1 infected patients with TB-IRIS are compared relative to data from saline controls. Data points represent median ±IQR, *p<0.05 Mann Whitney test. (TIF) [file ppat.1005469.s010.tif]

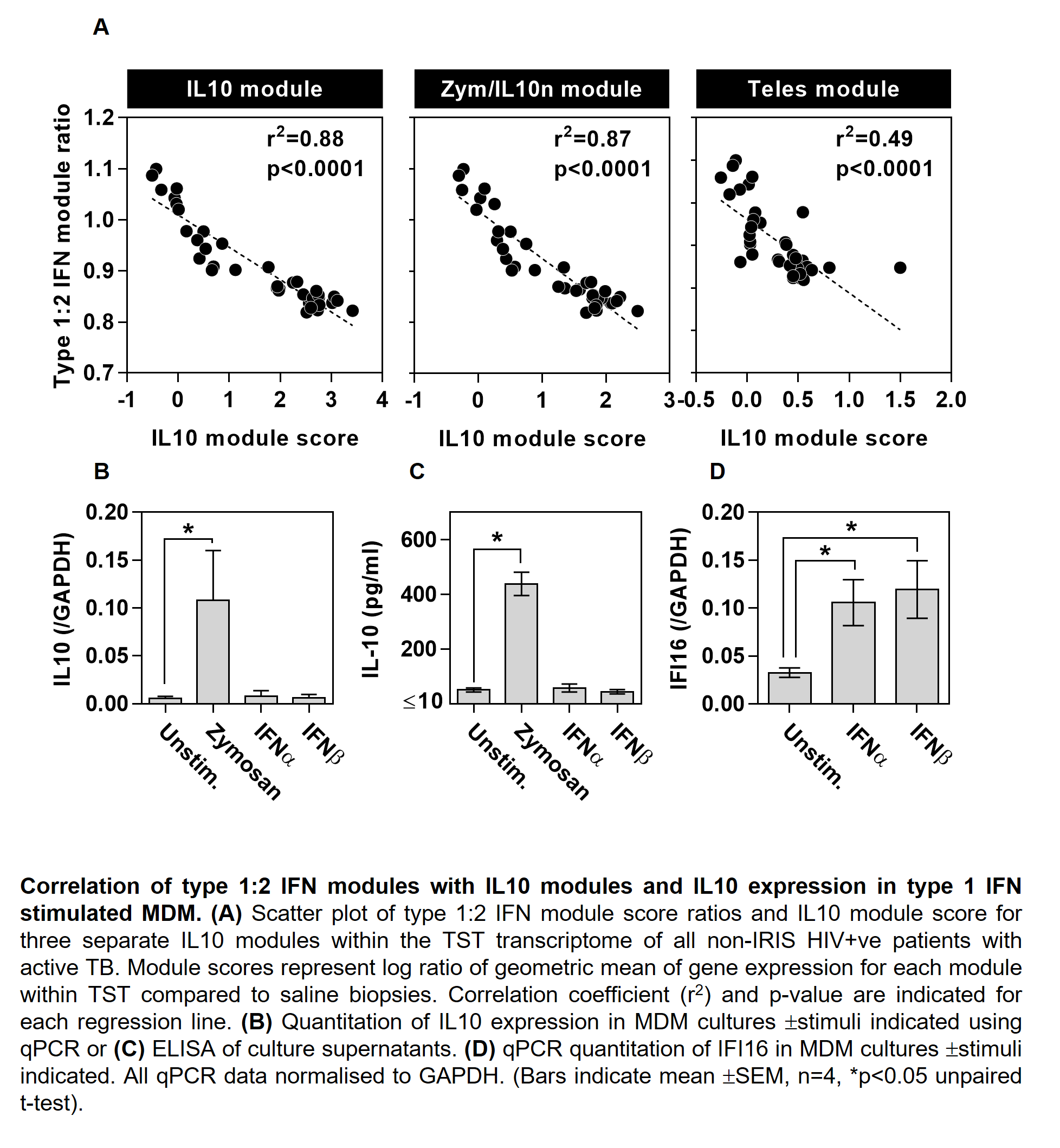

Supplement: S11 Fig — (A) Scatter plot of type 1:2 IFN module score ratios and IL10 module score for three separate IL10 modules within the TST transcriptome of all non-IRIS HIV+ve patients with active TB. Module scores represent log ratio of geometric mean of gene expression for each module within TST compared to saline biopsies. Correlation coefficient (r2) and p-value are indicated for each regression line. (B) Quantitation of IL10 expression in MDM cultures ±stimuli indicated using qPCR or (C) ELISA of culture supernatants. (D) qPCR quantitation of IFI16 in MDM cultures ±stimuli indicated. All qPCR data normalised to GAPDH. (Bars indicate mean ±SEM, n = 4, *p<0.05 unpaired t-test). (TIF) [file ppat.1005469.s011.tif]

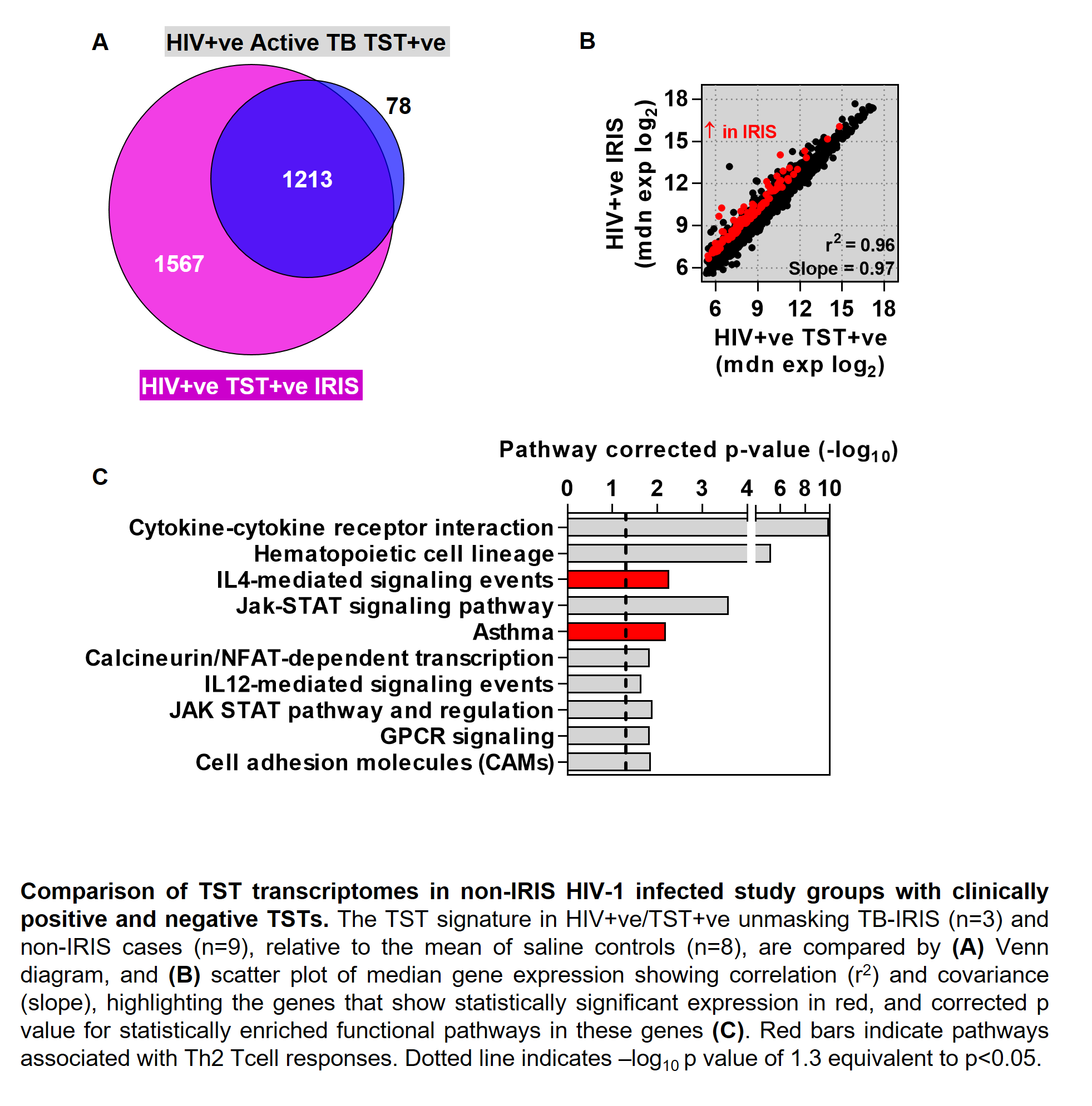

Supplement: S12 Fig — The TST signature in HIV+ve/TST+ve unmasking TB-IRIS (n = 3) and non-IRIS cases (n = 9), relative to the mean of saline controls (n = 8), are compared by (A) Venn diagram, and (B) scatter plot of median gene expression showing correlation (r2) and covariance (slope), highlighting the genes that show statistically significant expression in red, and corrected p value for statistically enriched functional pathways in these genes (C). Red bars indicate pathways associated with Th2 Tcell responses. Dotted line indicates–log10 p value of 1.3 equivalent to p<0.05. (TIF) [file ppat.1005469.s012.tif]
