# Supplementary material for: In Vivo Molecular Dissection of the Effects of HIV-1 in Active Tuberculosis
Source: PLoS Pathog. 2016 Mar 17;12(3):e1005469. doi: 10.1371/journal.ppat.1005469 (PMC4795555; doi:10.1371/journal.ppat.1005469)
Supplement: S1 Table — (DOCX) [file ppat.1005469.s013.docx]

### Table S1

Inclusion and exclusion criteria and description of study groups

| Inclusion criteria | Exclusion criteria | Study groups | |
| --- | --- | --- | --- |
| - Clinical ±microbiological diagnosis of active TB - >16 years age - <4 weeks since starting TB treatment | - Neoplastic disease - AIDS defining disease other than tuberculosis or KSHV infection - Hepatitis B/C co-infection - Immunomodulatory therapy (e.g. corticosteroids or interferon) - Immunization within preceding 2 weeks - Existing paradoxical reaction to anti-tuberculosis treatment - Previous keloid formation | HIV-ve | TST induration >10mm |
|  |  | HIV+ve | TST induration >10mm |
|  |  |  | TST induration <10mm |
|  |  |  | Started antiretroviral treatment 2-8 weeks before onset of active TB |
