# Supplementary material for: In Vivo Molecular Dissection of the Effects of HIV-1 in Active Tuberculosis
Source: PLoS Pathog. 2016 Mar 17;12(3):e1005469. doi: 10.1371/journal.ppat.1005469 (PMC4795555; doi:10.1371/journal.ppat.1005469)
Supplement: S2 Table — (DOCX) [file ppat.1005469.s014.docx]

### Table S2

Table of accession numbers for all array data within ArrayExpress

| Description of data set | Array express accession no | Reference DOI |
| --- | --- | --- |
| Expression profiles of TST biopsies in patients with active TB. | E-MTAB-3254 | N/A (First presentation in this manuscript) |
| Expression profiles of **TST biopsies in healthy individuals** with clinically positive and clinically negative TST responses. | E-TABM-1157 | 10.1002/eji.201141841 |
| Expression profiles of peripheral blood samples from patients with active TB and healthy volunteers. | E-MTAB-3260 | N/A (First presentation in this manuscript) |
| Expression profiles of MDM ±stimulation with Mtb. | E-TABM-1163 | 10.1093/infdis/jit621 |
| Expression profiles of MDM ±stimulation with LPS, IFNβ and IFNγ. | E-MEXP-2032 | 10.1097/QAD.0b013e328331a4ce |
| Expression profiles of MDM ±stimulation with Pam_2_CSK4 or *Streptococcus pneumonia*. | E-MTAB-1541 | 10.4049/jimmunol.1401413 |
| Expression profiles of MDM ±stimulation with IFNγ, TNFα, IL4/IL13, TGFβ/IL10, IL10 or zymosan. | E-MTAB-3255 | N/A (First presentation in this manuscript) |
| Expression profiles from a **variety of resting and activated human immune cells** used to derive cell type specific modules for T cells, NK cells, B cells, monocytes and neutrophils. | E-GEOD-22886 | 10.1038/sj.gene.6364173 |
| Expression profiles from a **variety of human immune cells** used to validate cell type specific modules for T cells, NK cells, B cells, monocytes and neutrophils. | E-GEOD-28490 | 10.1371/journal.pone.0029979 |
| Expression profiles of caseous human pulmonary TB granulomas derived from patients with active TB and healthy human lung samples. | E-GEOD-20050 | 10.1002/emmm.201000079 |
| Expression profiles of lung biopsies from idiopathic pulmonary fibrosis (IPF) or control lung tissue. | E-GEOD-53845 | 10.1136/thoraxjnl-2013-204596 |
| Expression profiles of clinically normal, non-lesional skin of psoriasis patients injected with IFNγ or saline. | E-GEOD-32407 | 10.1038/jid.2011.458 |
| Expression profile of endometrium in women treated with the progesterone receptor modulator asoprisnil (25mg) or placebo. | E-GEOD-47577 | 10.4049/jimmunol.1300958 |
| Expression profiles of mediastinal lymph node biopsies from patients with sarcoidosis and cancer. | E-MTAB-2547 | 10.1378/chest.15-0647 |
| Expression profiles of skin lesions of patients with erythema nodosum leprosum or lepromatous leprosy. | E-GEOD-16844 | 10.1086/650318 |
